# Pathophysiology of bone disease in chronic kidney disease: from basics to renal osteodystrophy and osteoporosis

**DOI:** 10.3389/fphys.2023.1177829

**Published:** 2023-06-05

**Authors:** Armando Aguilar, Laia Gifre, Pablo Ureña-Torres, Natalia Carrillo-López, Minerva Rodriguez-García, Elisabeth Massó, Iara da Silva, Víctor López-Báez, Maya Sánchez-Bayá, Águeda Prior-Español, Marina Urrutia, Javier Paul, Misael C. Bustos, Anna Vila, Isa Garnica-León, Juan F. Navarro-González, Lourdes Mateo, Jordi Bover

**Affiliations:** ^1^ Autonomous University of Chiapas, Tuxtla Gutiérrez, Mexico; ^2^ Department of Nephrology, Mexican Social Security, IMSS General Hospital of Zone No 2, Tuxtla Gutiérrez, Mexico; ^3^ Department of Rheumatology, Hospital Germans Trias i Pujol, Badalona (Barcelona), Catalonia, Spain; ^4^ AURA Saint Ouen, Department of Nephrology and Dialysis and Department of Renal Physiology, Necker Hospital, University of Paris Descartes, Paris, France; ^5^ Bone and Mineral Research Unit, Instituto de Investigación Sanitaria del Principado de Asturias (ISPA), Oviedo, Asturias, Spain; ^6^ Nephrology Clinical Management Unit, Central University Hospital of Asturias (HUCA), Oviedo, Asturias, Spain; ^7^ Department of Nephrology, University Hospital Germans Trias i Pujol (HGiTP), Badalona (Barcelona), Catalonia, Spain; ^8^ REMAR-IGTP Group, Research Institute Germans Trias i Pujol, Can Ruti Campus, Badalona (Barcelona), Catalonia, Spain; ^9^ Department of Nephrology, Pontificia Catholic University of Chile, Santiago, Chile; ^10^ Research Unit and Nephrology Service, University Hospital of Nuestra Señora de la Candelaria, Santa Cruz de Tenerife, Islas Canarias, Spain; ^11^ Instituto de Tecnologías Biomédicas, Universidad de la Laguna, Islas Canarias, Spain

**Keywords:** CKD-MBD, renal osteodystrophy, osteoporosis, adynamic bone disease, sclerostin, RANKL (receptor activator for nuclear factor k B ligand), parathyroid hormone, Wnt

## Abstract

Chronic kidney disease (CKD) is a highly prevalent disease that has become a public health problem. Progression of CKD is associated with serious complications, including the *systemic* CKD-mineral and bone disorder (CKD-MBD). Laboratory, bone and vascular abnormalities define this condition, and all have been independently related to cardiovascular disease and high mortality rates. The “old” cross-talk between kidney and bone (classically known as “renal osteodystrophies”) has been recently expanded to the cardiovascular system, emphasizing the importance of the bone component of CKD-MBD. Moreover, a recently recognized higher susceptibility of patients with CKD to falls and bone fractures led to important paradigm changes in the new CKD-MBD guidelines. Evaluation of bone mineral density and the diagnosis of “osteoporosis” emerges in nephrology as a new possibility “if results will impact clinical decisions”. Obviously, it is still reasonable to perform a bone biopsy if knowledge of the type of renal osteodystrophy will be clinically useful (low *versus* high turnover-bone disease). However, it is now considered that the inability to perform a bone biopsy may not justify withholding antiresorptive therapies to patients with high risk of fracture. This view adds to the effects of parathyroid hormone in CKD patients and the classical treatment of secondary hyperparathyroidism. The availability of new antiosteoporotic treatments bring the opportunity to come back to the basics, and the knowledge of new pathophysiological pathways [OPG/RANKL (LGR4); Wnt-ß-catenin pathway], also affected in CKD, offers great opportunities to further unravel the complex physiopathology of CKD-MBD and to improve outcomes.

## Introduction

Chronic kidney disease (CKD) is a highly prevalent and progressive condition that affects more than 10% of the general population around the globe (Kalantar-Zadeh et al., 2021; Csaba, 2022). CKD has also emerged as one of the leading causes of morbidity and mortality and represents significant challenges for healthcare systems and societies worldwide (Elshahat et al., 2020; Kalantar-Zadeh et al., 2021; Alberto Ortiz, 2022; Csaba, 2022). Importantly, progression of CKD is associated with a number of serious complications, including mineral metabolism disorders and bone pathology, and these are independently associated with fractures, accelerated vascular calcification, cardiovascular disease, and dismal outcomes (Moe et al., 2006; Kidney Disease: Improving Global Outcomes KDIGO CKD-MBD Update Work Group, 2017; Wang et al., 2018; Kidney Disease: Improving Global Outcomes KDIGOCKD–MBD Work Group, 2009). Bone is no longer regarded simply as an organ that supports and protects internal organs and we even considered to be as a new endocrine organ at the heart of the CKD-mineral and bone disorders (CKD-MBD) (Vervloet et al., 2014). Bone is capable of secreting countless hormones and molecules essential for the normal physiology of many other body systems (Vervloet et al., 2014). Consequently, CKD-MBD is a currently accepted term which refers to a *systemic* disorder of mineral and bone metabolism due to CKD manifested by various laboratory, bone, and vascular abnormalities (Moe et al., 2006; Kidney Disease: Improving Global Outcomes KDIGO CKD-MBD Update Work Group, 2017; Kidney Disease: Improving Global Outcomes KDIGOCKD–MBD Work Group, 2009; Torregrosa et al., 2022).

Previously, the term *renal osteodystrophy* (ROD) had been coined in 1943 (Llach et al., 2000), 60 years after the identification of an association between bone disease and kidney failure (Lucas, 1883; Llach et al., 2000). ROD was a very broad term that classically included all the skeletal manifestations in patients suffering from CKD or end-stage kidney disease (CKD G5D) (Llach et al., 2000). In children, rickets and skeletal deformities were also included, while osteosclerosis and osteoporosis (OP) were globally considered less common (Llach et al., 2000). Nevertheless, ROD is nowadays considered to be only one component of the wider complex CKD-MBD after a histomorphometric analysis of a bone biopsy has been performed (Moe et al., 2006; Kidney Disease: Improving Global Outcomes KDIGO CKD-MBD Update Work Group, 2017). Derangements induced by CKD are multiple, ranging from the classically described disturbances of vitamin D metabolism, calcium, and phosphate balance, through increased levels of parathyroid hormone (PTH) (secondary hyperparathyroidism) to the more recently recognized increases in fibroblast growth factor 23 (FGF23) and sclerostin, or decreased serum klotho levels, among others (Kidney Disease: Improving Global Outcomes KDIGO CKD-MBD Update Work Group, 2017; Llach et al., 2000; Hruska et al., 2017). Chronic metabolic acidosis, the use of drugs such as prednisone or calcineurin inhibitors (used to treat certain kidney diseases), diabetes mellitus, accelerated aging, female gender, and early menopause can additionally affect one or more bone properties. A detailed description of all the pathophysiological pathways leading to different forms of ROD is beyond the scope of this article, and we refer interested readers to excellent reviews elsewhere (Goltzman et al., 2018). Nevertheless, in this narrative review we will briefly address the relevant basics as well as evolving topics in bone pathophysiology of interest beyond nephrology. In fact, important paradigm changes from ROD to OP have occurred in recent CKD-MBD guidelines and these need to be more widely known.

## Bone cells

The most important cells of bone tissue are osteoblasts (OBs), osteoclasts (OCs), osteocytes, and bone-lining cells.

a) OBs develop from pluripotential mesenchymal stem cells (MSCs). MSCs can differentiate into adipocytes, chondrocytes, myocytes, or OBs depending on the transcription factor acting on them. Bone morphogenic proteins (BMPs) and the Wnt signaling pathway are related to OB differentiation. The *canonical* Wnt signaling pathway induces transcription factors that favor OB differentiation, and the *non-canonical* Wnt pathway inhibits the differentiation of MSCs to other cell types, resulting overall in a positive balance towards OB formation. The main function of OBs is the *formation* of the bone matrix through the synthesis and secretion of type 1 collagen and other non-collagenous proteins which will later be mineralized. OBs also collaborate in this function by releasing phosphate contained in their vesicles and, together with the calcium and phosphate contained in the extracellular fluid, compose the main mineral of cortical bone (calcium hydroxyapatite crystals) (Day et al., 2005; Takada et al., 2007; Guo et al., 2010). The N-terminal propeptide of type I procollagen (P1NP) has been identified by the International Osteoporosis Foundation (IOF) and the International Federation of Clinical Chemistry (IFCC) to be one of the reference markers of bone turnover (formation) for fracture risk prediction and monitoring of OP treatment (Vasikaran et al., 2011). It is important to take into account the fact that only the measurement of *intact* P1NP is not affected by the decreased renal function in patients with CKD (Bover et al., 2021a; Tridimas et al., 2021). Alkaline phosphatase (AP, especially the bone isoform) can also be used to evaluate bone turnover in CKD (Bover et al., 2021a). Actually, AP can reflect not only OB activity in bone but also OB-like cell activity in the active process of *ossification* of vascular smooth muscle cells (Bover et al., 2018; Bover et al., 2021b).
b) OCs derive from precursor cells of the monocyte-macrophage lineage. OC differentiation and survival require the presence of molecules such as the macrophage colony-stimulating factor (M-CSF) and the important receptor activator of NF-κB ligand (RANKL). OB-synthesized osteoprotegerin (OPG) acts as a high-affinity decoy receptor for RANKL, inhibiting RANKL action on the OC-RANK receptor (Wada et al., 2006). The ratio between RANKL and OPG determines the degree of osteoclastic differentiation (Gori et al., 2000), although blood measurements are not of clinical use. It has been recently described another RANKL receptor, the leucine-rich repeat-containing G-protein-coupled receptor 4 (LGR4), which competes with RANK to bind RANKL and suppresses canonical RANK signaling during OC differentiation (Luo et al., 2016). It also regulates OB differentiation *in vivo* and *in vitro* (Luo et al., 2009). LGR4 is also present in different tissues and consequently it has been linked with systemic roles from development to metabolic regulation (Filipowska et al., 2022).

The main function of the OC is bone *resorption*. OCs must be activated by binding to the bone matrix, polarizing and forming podosomes and different membrane domains (the sealing zone, the characteristic ruffled border, and the functional secretory domain). Each of these domains is extremely important for bone resorption, collagen degradation, and the return of calcium and phosphate to the bloodstream (Luxenburg et al., 2007). Lysosomal enzymes derived from OCs are responsible for breakdown of the collagenous bone matrix at specific sites (Bover et al., 2021a). Resultant products such as carboxy-terminal crosslinking telopeptide of type 1 collagen (CTX) are considered reference markers for bone resorption in the general population (Wheater et al., 2013). However, CTX is highly dependent on kidney function; therefore, the use of CTX cannot be recommended in patients with CKD (Bover et al., 2021a). For this reason, tartrate-resistant acid phosphatase 5b (TRAP5b) is gaining increasing importance, given that its concentration is not kidney dependent (Bover et al., 2021a).

c) Osteocytes represent 95% of all bone cells. These cells are mature OBs that occupy the lacunar space and are surrounded by the unmineralized osteoid matrix. After mineralization, these buried cells become osteocytes and acquire long dendritic-like processes, giving them a star-shaped appearance. Dendritic processes extend along the canaliculi in the bone matrix, interacting with other osteocytes or with OBs on the surface. Osteocytes have a position that allows detection of both mechanical and metabolic signals and act accordingly, directly activating OBs and indirectly OCs, thus initiating the classic remodeling cycle. Osteocytes influence OBs in two directions, either upregulating them through the production of messengers such as nitric oxide and prostaglandin E2 or downregulating them through the secretion of sclerostin (Rochefort et al., 2010). As we will discuss later, osteocytes and sclerostin have gained increased attention in bone pathophysiology, nephrology, and medicine in general since their discovery and the development of new treatments for bone diseases such as OP. Osteocytes are also the main source of FGF23, a pleiotropic hormone responsible of suppressing phosphate reabsorption and calcitriol synthesis in the kidney (Orlando, 2020). Although FGF23 monitoring is not yet included in the regular management of CKD-MBD, it is important to emphasize its role in the development of left ventricular hypertrophy (Richter and Faul, 2018), among other systemic effects (Vervloet, 2020), and its powerful inverse association with survival in CKD patients (Gutiérrez et al., 2008).

## Normal bone anatomy and physiology

Two histologically different regions can be distinguished in bone: a) *cortical or compact bone*, which represents up to 80% of the skeleton, is composed mainly of calcium hydroxyapatite, and has the main function of providing mechanical support, and b) *trabecular or cancellous bone,* which is less abundant, is mainly composed of an organic matrix rich in type 1 collagen, has an important endocrine function, and contains the bone marrow.

Several biological processes occur in bone tissue during life. Bone undergoes *modelling and remodelling* in order to grow or change shape (Katsimbri, 2017). Bone *modelling* is a process by which bones change shape or size in response to physiological influences or mechanical forces that are encountered by the skeleton (Katsimbri, 2017). *Remodelling* is the process which allows bone to maintain its mineral homeostasis and strength (Katsimbri, 2017). Once bone growth stops when adult age is reached (after bone formation and shaping), the bone tissue requires dynamic remodelling to maintain adequate resistance and properties (Katsimbri, 2017). Old bone tissue is removed and replaced by new tissue through an organized process occurring within temporary anatomical structures described as basic multicellular units. Five stages are described in the remodelling process. They are widely known as activation, resorption, rest or reversal, bone formation, and termination; however, a detailed description of these mechanisms is beyond the scope of this review (Burger et al., 2003; Martin and Sims, 2005; Bonewald, 2007; Bonewald and Johnson, 2008; Katsimbri, 2017).

### Metabolic bone biopsy

Bone dynamics can only be assessed by a *bone biopsy*, a classic procedure previously performed frequently by nephrologists in order to precisely distinguish among different forms of ROD (e.g., high and low-turnover bone disease) (Moe et al., 2006; Bover et al., 2021a). No biomarker or imaging studies can match this gold standard (Bover et al., 2021a). Tetracycline labeling allows *dynamic* quantification, e.g., by analyzing the important *bone formation rate* and the *mineral apposition rate*, among other dynamic parameters. Many other *static* evaluations, such as the *osteoid area* or the percentage of *fibrosis,* contribute to a precise diagnosis (Mazzaferro and Pasquali, 2021). Essentially, bone biopsies should evaluate bone turnover, mineralization and volume (following the useful acronym TMV) (Moe et al., 2006). Thus, different patterns of ROD are usually described (see below): high-turnover osteitis fibrosa or mild hyperparathyroidism, low-turnover adynamic bone disease (ABD) and osteomalacia, and the mixed form named uremic osteodystrophy (Moe et al., 2006).

Bone biopsies are regaining importance in nephrology, especially with the paradigm changes that have appeared in recent CKD-MBD guidelines (Kidney Disease: Improving Global Outcomes KDIGO CKD-MBD Update Work Group, 2017; Torregrosa et al., 2022). The potential need for better understanding of the consequences of the current more aggressive use of anti-OP treatments in patients with CKD represents an additional reason for this trend (Kidney Disease: Improving Global Outcomes KDIGO CKD-MBD Update Work Group, 2017; Torregrosa et al., 2022). Thus, it is currently considered reasonable to perform a bone biopsy if knowledge of the type of ROD will impact treatment decisions in patients with CKD G3a-G5D (not graded) (Kidney Disease: Improving Global Outcomes KDIGO CKD-MBD Update Work Group, 2017; Torregrosa et al., 2022). It was previously considered reasonable to perform a bone biopsy “in various settings including, but not limited to: unexplained fractures, persistent bone pain, unexplained hypercalcemia, unexplained hypophosphatemia, possible aluminum toxicity, and prior to therapy with bisphosphonates in patients with CKD-MBD” (not graded either) (Kidney Disease: Improving Global Outcomes KDIGOCKD–MBD Work Group, 2009). A wider description of indications and the technical procedure is beyond the scope of this review and readers are referred to literature elsewhere (Salusky et al., 1988; Torres et al., 2014; Evenepoel et al., 2017). However, it should be emphasized that efforts are currently being made to standardize the variable “normality” values used in different laboratories when performing histomorphometric analysis (Sprague et al., 2016a). It is also being suggested that bone mineral density (BMD) testing should be used to assess fracture risk “if results will impact treatment decisions” in patients with CKD G3a-G5D with evidence of CKD-MBD and/or risk factors for OP (see later) (Kidney Disease: Improving Global Outcomes KDIGO CKD-MBD Update Work Group, 2017; Torregrosa et al., 2022), since bone biopsy is not useful for fracture risk prediction.

## Effects of parathyroid hormone on bone tissue

PTH plays a very important role in the dynamics of bone tissue. Several unanswered but important questions remain about the skeletal actions of PTH, with differences between intermittent administration and constant exposure to high levels (Hock and Gera, 1992; Rendina-Ruedy and Rosen, 2022). Thus, constant high PTH levels can increase bone remodelling to exert a catabolic effect on cortical and, to some extent, trabecular bone (Goltzman, 2018). On the other hand, intermittent administration of PTH can exert an anabolic effect on bone; this is especially the case for trabecular bone but also to some extent for cortical bone (Goltzman, 2018). We describe below some of the effects of PTH on bone cells and the remodelling stages.

### PTH and osteoblasts

**FIGURE 1:**
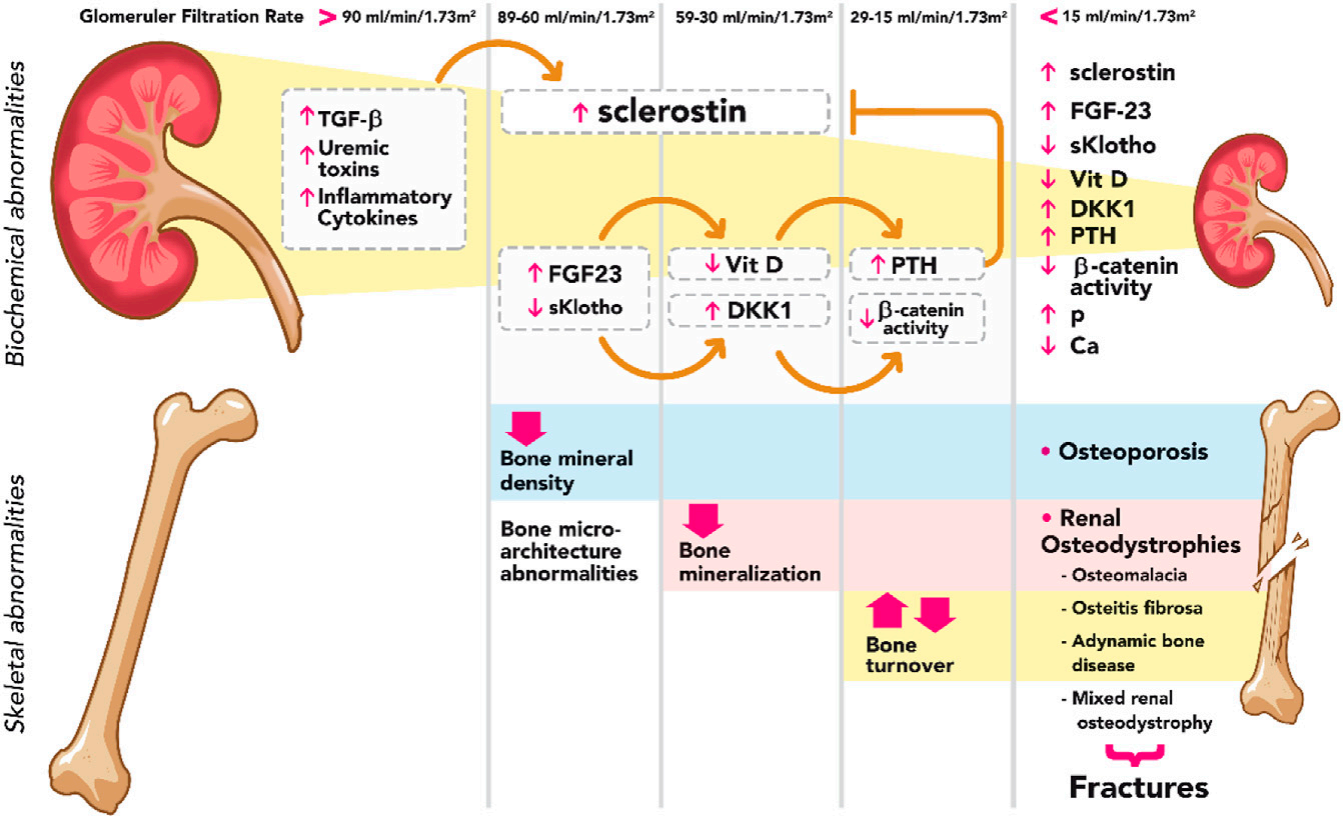
Summary diagram of biochemical and skeletal abnormalies in CKD. Sclerostin rises from very early stages of CKD with TGF-β, uremic toxins, and inflammatory cytokines as main known stimuli. Elevated FG23, in the presence of sKlotho, increases the expression of Dkk1, a Wnt inhibitor; consequently, Wnt-β catenin signaling remains suppressed during the course of CKD. FGF23 decreases the levels of calcitriol, resulting in a stimulus for PTH synthesis and secretion which, on the other hand, decreases the effects of sclerostin. TGF-β, transforming growth factor beta; FGF-23, fibroblast growth factor-23; Vit D, vitamin D; DKK1, Dickkopf 1; PTH, parathyroid hormone; P phosphorus; Ca calcium.

PTH administration enhances bone formation by inducing transcriptional changes in several OB pathways, being the via adenyl cyclase and protein kinase A (PKA) the most prominent (Rendina-Ruedy and Rosen, 2022) (Figure 1). PTH also influences the entire life cycle of OBs, from their differentiation from pluripotent MSCs through to activation and even apoptosis. PTH appears to increase the amount of OB precursors in the bone marrow through a direct action. The bone marrow cells capable of differentiating to OBs are the colony-forming units-fibroblast (CFU-F) and old studies already demonstrated that the administration of PTH (Kalantar-Zadeh et al., 2021; Csaba, 2022; Alberto Ortiz, 2022; Elshahat et al., 2020; Moe et al., 2006; Kidney Disease: Improving Global Outcomes KDIGO CKD-MBD Update Work Group, 2017; Wang et al., 2018; Kidney Disease: Improving Global Outcomes KDIGOCKD–MBD Work Group, 2009; Vervloet et al., 2014; Torregrosa et al., 2022; Llach et al., 2000; Lucas, 1883; Hruska et al., 2017; Goltzman et al., 2018; Day et al., 2005; Takada et al., 2007; Guo et al., 2010; Vasikaran et al., 2011; Tridimas et al., 2021; Bover et al., 2021a; Bover et al., 2018; Bover et al., 2021b; Wada et al., 2006; Gori et al., 2000; Luo et al., 2016; Luo et al., 2009; Filipowska et al., 2022; Luxenburg et al., 2007; Wheater et al., 2013; Rochefort et al., 2010; Orlando, 2020; Richter and Faul, 2018; Vervloet, 2020; Gutiérrez et al., 2008) to rats for 1 week resulted in the doubling of CFU-F compared with placebo-treated rats (Nishida et al., 1994).

It is well known that PTH cross-talks with the cellular Wnt signaling pathway, stimulating bone formation by increasing the number of OBs. PTH also reduces the levels of sclerostin (downregulating the SOST gene), thus providing another paracrine mechanism through which PTH can stimulate the differentiation of OBs (Bellido et al., 2005; Loots et al., 2005; Drake et al., 2010; Nagata et al., 2022). Within OBs, PTH stimulates the formation of a tertiary complex PTH/PTHrp receptor and the Wnt co-receptor LRP6 (Wan et al., 2008), highlighting the importance of this complex since mice lacking LRP6 in OBs do not respond to iPTH (Wan et al., 2008). This signaling link between PTH and Wnt has also been strengthened by the observation that PTH reduces other Wnt inhibitors [such as Dickkopf 1 (Dkk1), secreted frizzled-related proteins (Sfrp) 1 and 4] (Carrillo-López et al., 2016), and that inhibition of Wnt signaling by Dkk1 prevents the effects of PTH on bone (Li et al., 2006). It has recently been reported that a newly identified osteogenic growth factor, osteolectin/Clec11a, is required for the maintenance of skeletal bone mass during adulthood, and that the combined administration of osteolectin and PTH, but not osteolectin and sclerostin inhibitor, additively increases bone volume (Zhang et al., 2021). These results demonstrate that PTH promotes osteolectin expression and that osteolectin mediates at least part of the effect of PTH on bone formation. (Zhang et al., 2021).

PTH also affects other important series of signaling pathways. For example, PTH stimulates Runx2, an essential transcription factor in bone required for OB differentiation (Arumugam et al., 2019). PTH stimulates the synthesis of growth factors including insulin-like growth factor (IGF)-1 and FGF, both of which are required for the anabolic effects of iPTH (Bikle et al., 2002; Hurley et al., 2006; Wang et al., 2007). Another PTH target gene that has been extensively studied in OBs is the matrix metalloproteinase 13 (MMP13) gene, the expression of which is mediated through an intricate signaling pathway involving PKA, Runx2, sirtuin-1, and others. Thus, PTH upregulation of MMP13 plays an important role in how OBs remodel old bone matrix as they synthesize new type I collagen. (Shimizu et al., 2010; Shimizu et al., 2014; Fei et al., 2015). PTH has also been shown to induce T lymphocytes in the bone marrow microenvironment to produce cytokines that stimulate the differentiation of OBs (Terauchi et al., 2009). Finally, one of the most important effects of PTH in OBs is inhibition of OB apoptosis (Allan et al., 2008; De Pasquale et al., 2008). All these positive actions of PTH on OBs and bone formation represent the basis on which today recombinant PTH (teriparatide) constitutes an alternative in the treatment of OP (Neer et al., 2001). Abaloparatide (an analog of human PTH-related protein) has also recently been approved for OP treatment (Paul et al., 2016). Occasionally, teriparatide has also been used in the treatment of ABD in CKD patients (Cejka et al., 2010; Sumida et al., 2016).

### PTH and bone lining cells and osteocytes

Bone lining cells and osteocytes have properties which suggest that they belong to the OB lineage, expressing many of their genes. It has been shown that PTH activates these lining cells, inhibits osteocyte apoptosis, delays the differentiation of OB to lining cells, and increases the conversion of lining cells to OBs (Jang et al., 2016). Osteocytes express receptors for PTH on their surface, in such a way that their morphology and function, including cell retraction, mitochondrial congestion, and cell death, seem PTH regulated (Heller et al., 1950; Cameron et al., 1967; O’Brien et al., 2008; Rhee et al., 2011). On the other hand, PTH upregulates osteocytic RANKL, and RANKL plays a critical role in the PTH-induced increases in bone resorption (see below) (Nakashima and Takayanagi, 2011; Xiong et al., 2011; Ben-awadh et al., 2014; Xiong et al., 2014).

### PTH and osteoclasts

OCs do not express receptors for PTH; therefore, PTH action is indirectly mediated through OBs. M-CSF and RANKL are the two main cytokines that drive OC differentiation and function (Feng and Teitelbaum, 2013), and PTH has been shown to increase the expression of these molecules (Itoh et al., 2000). In fact, there are multiple cellular sources of these two cytokines in bone (hypertrophic chondrocytes, marrow stromal cells, osteoblasts, resident marrow lymphocytes, and osteocytes) (O’Brien et al., 2013), and RANKL is a well-studied PTH target gene in multiple cell types (Fu et al., 2002; Fu et al., 2006; Kim et al., 2006; Kim et al., 2007). During OC-mediated bone resorption, growth factors such as TGF-ß1 and IGF-1 are released. IGF-1 is maintained in the bone matrix in complex with binding proteins (IGFBP) and OC bone remodelling leads to IGFBP cleavage and subsequent IGF-1 release (Crane and Cao, 2014). Finally, recent translation studies highlight the potent amplificatory action of T-cell on PTH-induced bone resorption in parathyroid disease (Neale, 2017). PTH acts on CD4^+^ T-cell to drive up TNFα and IL-17, further amplifying osteoblastic RANKL production and down-regulating OPG, establishing favorable conditions for osteoclastic bone resorption (Neale, 2017).

## Disorders of bone remodelling in CKD

Despite previous descriptions of “late rickets associated with albuminuria” by Lucas in The Lancet in 1883 and “tumor of the parathyroid gland” by MacCallum in 1905 (Llach et al., 2000), it was not until 1924 that a study of a patient with severe bone demineralization and multiple fractures led to the discovery that the disease resided in the parathyroid gland (Albright and Reifenstein, 1948). In 1925 the first resection of a parathyroid adenoma was performed (Albright and Reifenstein, 1948)**.** It 1933 Langmead suggested for the first time that parathyroid hyperplasia was secondary to advanced CKD (Llach et al., 2000). Other patients began to be diagnosed with this new disease and there was a need to study derangements in divalent ion metabolism, vitamin D, and the molecule produced by the parathyroid glands that caused so much damage to the bone tissue (Albright and Reifenstein, 1948; Handler et al., 1954; Llach et al., 2000). In the 1960s, Stanbury and Lamb as well as Dent and co-workers (Llach et al., 2000) linked abnormalities of divalent ion metabolism, PTH, and vitamin D with the bone abnormalities observed in CKD. With the advent of radioimmunoassays for PTH (Brewer and Ronan, 1970; Niall et al., 1970; Llach et al., 2000), high circulating levels of PTH were detected at earlier stages of CKD (Llach et al., 2000); however, it was not until 1970 that characterization of the PTH molecule was completed, which helped to its cloning in 1983 (Brewer and Ronan, 1970; Niall et al., 1970; Vasicek et al., 1983). In the 1990s, highly sensitive immunoassays were developed and its receptor was finally cloned in 1991 (Jüppner et al., 1991; Abou-Samra et al., 1992). We now know that at least very low or very high levels of PTH (i.e., less than 2X or more than 9X the upper normal limit for the used assay) are associated with low or high-turnover bone disease (ABD or osteitis fibrosa, respectively) in dialysis patients; both extremes increase not only the risk of fractures but also mortality by different means (Kidney Disease: Improving Global Outcomes KDIGO CKD-MBD Update Work Group, 2017; Kidney Disease: Improving Global Outcomes KDIGOCKD–MBD Work Group, 2009; Torregrosa et al., 2022).

It was considered previously that the elevation of PTH was the main responsible for skeletal abnormalities in CKD; however, recent evidence has shown that changes in bone tissue occur from early stages (Sabbagh et al., 2012; Baron and Kneissel, 2013a). The increase in sclerostin and FGF23 levels, two molecules secreted by osteocytes, and the consequent repression of Wnt-β catenin signaling pathway represent a clear mechanistic example explaining the impairment of bone health from the onset of CKD (Figure 1) (Cejka et al., 2012; Moysés and Schiavi, 2015; Drüeke and Massy, 2016). Increasing PTH, to a certain extent, may thus appear as an adaptive mechanism to maintain not only normal serum calcium, phosphate and/or calcitriol levels but also a normal bone remodeling (Torregrosa et al., 2022). Another molecule that is elevated in the early stages of CKD and causes changes in bone dynamics is activin A (ActA), a member of the TGF-β superfamily that is secreted by renal fibroblasts. ActA activates receptors on the surface of OCs, leading to the activation of the intracellular protein Smad2. ActA also increases the expression of RANKL by OBs which binds to RANK on the surface of OCs, stimulating the expression and phosphorylation of c-Fos. Together with smad2, these molecules form a complex that enters the nucleus of OCs generating osteoclastogenesis and bone resorption (AgapovaFangSugatani et al., 2016; Sugatani et al., 2017; Cianciolo et al., 2021).

PTH also alters metabolic homeostasis through its actions in other cells and tissues, and myriad non-skeletal metabolic or anabolic effects have been attributed to PTH (Rendina-Ruedy and Rosen, 2022). However, in addition PTH has been considered a uremic toxin in CKD patients with secondary hyperparathyroidism because of many pleiotropic detrimental effects that can be attributed to this molecule (Ureña-Torres et al., 2018). Surgical parathyroidectomy and calcimimetics seem to reverse some of these untoward effects (Massy et al., 2014; Komaba et al., 2015; McMahon et al., 2015; Komaba et al., 2022).

Regarding bone, PTH levels are actually only indirectly associated with bone formation (a secondary impact), and they probably represent parathyroid activity at a certain time point much better than bone dynamics (Pablo and Covic, 2020). Moreover, unlike most other biomarkers or regulators of bone turnover, such as APs or P1NP, PTH secretion is not dictated by local bone demands (triggered on osteocytes via mechanical stimuli) (Bover et al., 2018). Actually, the major determinants of PTH synthesis and secretion are calcium, phosphate, vitamin D, and FGF23 levels (Levin et al., 2007; Sturgeon et al., 2017). In fact, according to current guidelines, the measurement of both serum PTH and bone AP can be used to evaluate bone disease in CKD patients because markedly high or low values predict underlying bone turnover (Kidney Disease: Improving Global Outcomes KDIGO CKD-MBD Update Work Group, 2017; Jørgensen et al., 2022a). Combination of PTH and APs significantly increases both the sensitivity and the specificity of the histopathological diagnosis (Ureña et al., 1996; Bover et al., 2018). These biomarkers offer the possibility of more frequent serial measurements that can guide us on therapeutic decision-making and follow-up. However, despite it has been recently recognized that diagnostic performance of biochemical markers of bone turnover is acceptable, with clinical utility in ruling out both high and low turnover bone disease, biomarkers do have some limitations such as the scarcity of data available to consider precise target figures, their biological variability and the inherent disparity of trials leading to different reference ranges and cut-offs (Jørgensen et al., 2022a; Jørgensen et al., 2022b).

While the parathyroid glands can be affected by a primary disease, such as parathyroid adenoma or the rare parathyroid neoplasms, *secondary* (or even tertiary) hyperparathyroidism is frequently observed during the course of CKD (Kidney Disease: Improving Global Outcomes KDIGO CKD-MBD Update Work Group, 2017; Torregrosa et al., 2022). Below we describe the two most common histologic patterns, which derive from very high (osteitis fibrosa) and relatively low levels of PTH (ABD).

## Osteitis fibrosa

**FIGURE 2:**
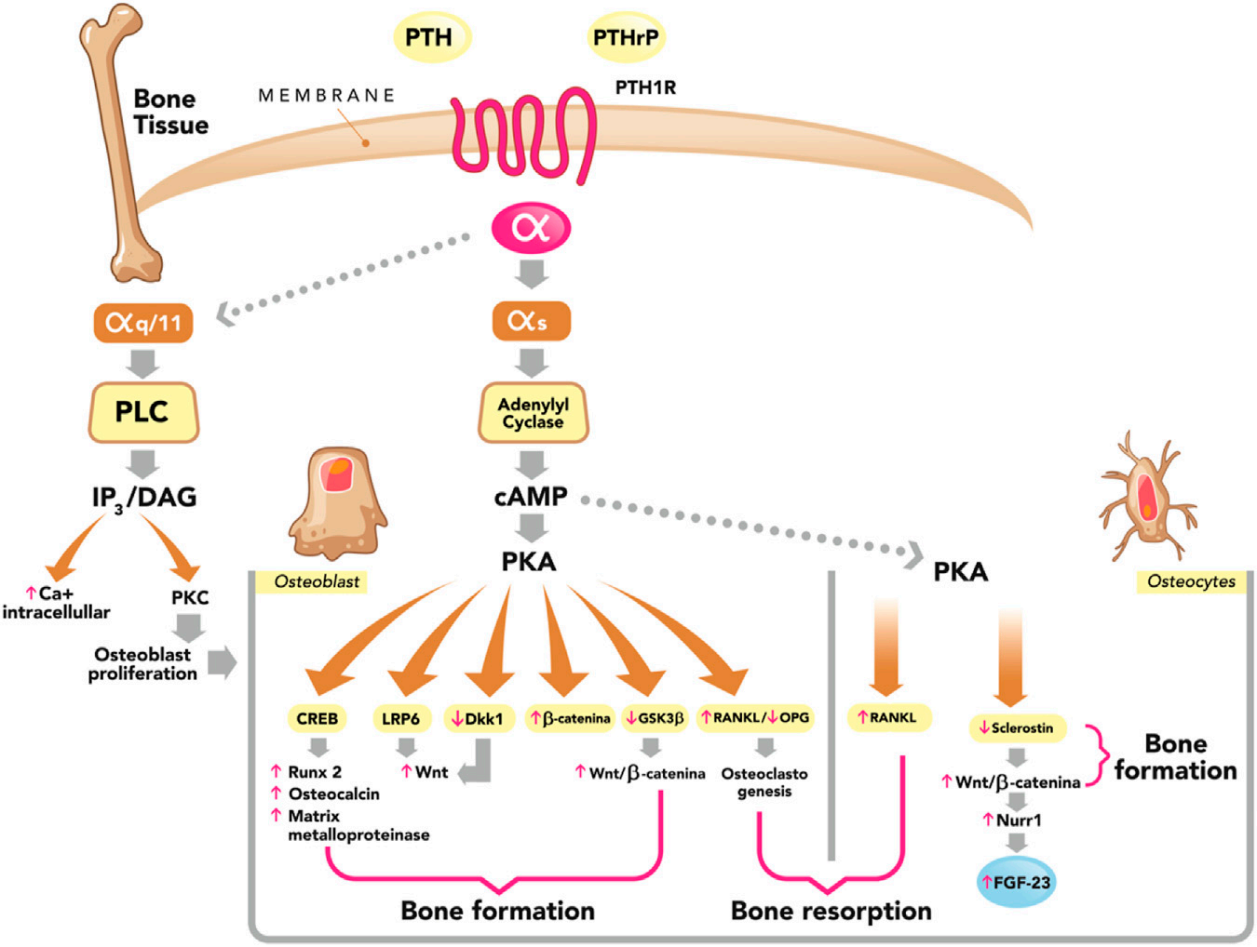
Dual effect of PTH on bone (formation and resorption). Parathyroid hormone (PTH) and PTH-related peptide (PTHrP) bind to the PTH/PTHrP type 1 receptor (PTH1R) with similar effects. This G protein-coupled receptor generates a dissociation of the α subunit. The most studied is the αs subunit and its main effector is adenylyl cyclase, which catalyzes the synthesis of the second messenger cyclic adenosine monophosphate (cAMP), which activates the cAMP-dependent protein kinase (PKA) pathway, phosphorylating several proteins that have diverse effects on bone and that we describe below: phosphorylation of cAMP response element-binding protein (CREB), increasing the expression of osteoblast-specific genes such as Runx 2, Osteocalcin and matrix metalloproteinase; stimulation of the Wnt-signaling pathway Wnt by means of the low-density lipoprotein receptor-related protein 6 (LRP6); phosphorylation and stabilization of β-catenin, suppression of Dickkopf 1 (DKK1) and glycogen synthase kinase 3 beta (GSK3β). Both are Wnt inhibitors, leading to the stimulation of the Wnt/B-catenin signaling pathway. All the aforementioned actions of PTH represent stimuli for bone formation (osteoanabolic effect). On the other hand, PTH in osteoblasts and osteocytes stimulates the production of receptor activator of nuclear factor Kβ ligand (RANKL) and decreases the production of osteoprotegerin (OPG), generating osteoclastogenesis and thereby stimulating bone resorption. In osteocytes, PTH suppresses the expression of sclerostin and this stimulates the Wnt/B-catenin signaling pathway, stimulating bone formation. PTH in osteocytes also stimulates the expression of nuclear receptor-related 1 protein (Nurr1), leading to an increase in the production of fibroblast growth factor-23 (FGF-23). PTH1R, in addition to coupling to αs, can also couple to αq/11, which activates the phospholipase C (PLC) by catalyzing the synthesis of its second messengers, inositol 1,4,5-trisphosphate (IP3); and diacylglycerol (DAG). In turn, PTH increases intracellular Ca2+ and activates protein kinase C (PKC) isozymes, PLC–PKC signaling pathway which is essential for osteoblast proliferation and for bone modeling and remodeling. Adapted from M. Bastepe, S. Turan and Q He (J Molec Endocrinol 2017; 58, R203-224).

The first report of this disease derived from very high PTH levels was made by von Recklinghausen in 1891 (Von Recklinghausen, 1891), but the full report of *“osteitis fibrosa cystica”* was not provided until 1936, by Albright and co-workers (Llach et al., 2000). CKD-associated hyperparathyroidism was considered by far the most prevalent form of ROD until recently, especially among advanced CKD and dialysis patients. However, current patterns show a lower prevalence ranging from 20% to 40% (Bover et al., 2014). The pathophysiology of secondary/tertiary hyperparathyroidism is beyond the scope of this review and interested readers are referred elsewhere (Llach et al., 2000; Cunningham et al., 2011; Lunyera and Scialla, 2018). After increased synthesis and secretion of PTH by multiple stimuli (hypocalcemia, hyperphosphatemia, decreased calcitriol, *etc.*), PTH binds to its receptors in bone tissue (mostly PTHR1), which are located on the surface of OBs and osteocytes. PTHR1 belongs to the B-family of G protein-coupled receptors, and once the PTH molecule has bound to its receptor, it triggers a series of intracellular signaling events (Figure 2).

In OBs, binding of PTH to its receptor induces the production of M-CSF and RANKL, responsible for the differentiation and activation of OCs (Kondo et al., 2002; Feng and Teitelbaum, 2013). PTH also induces the production of osteocytic RANKL, potentiating the differentiation and activation of OCs (Nakashima and Takayanagi, 2011; O’Brien et al., 2013; Ben-awadh et al., 2014; Xiong et al., 2014). The persistent and constant elevated PTH increases the bone resorption units, resulting in a considerable increase in resorptive areas and leading to negative bone balance (Malluche et al., 1982; Ma et al., 2001; Silva and Bilezikian, 2015). Increased osteoblastic activity leads to an unparalleled increase in the production of osteoid by OBs. Nevertheless, this osteoid does not have an orderly and laminar disposition, as in normal bone. This is known as *woven* bone, and this increase in osteoid is known as *hyperosteoidosis,* which occurs as a consequence of increased remodelling and not due to a delay in mineralization, as is the case with osteomalacia (Malluche et al., 1982; Feng and McDonald, 2011; Hruska et al., 2017).

Furthermore, in hyperparathyroidism there is activation of fibroblast-type mesenchymal cells that give rise to peritrabecular fibrosis (Lotinun et al., 2005; Lowry et al., 2008). The overall results is a loss of cortical bone secondary to the accelerated resorption, which far exceeds bone formation, and in the place of a laminar osteoid, a fibrous tissue even containing cysts is present (Naji Rad and Deluxe, 2022).

## Adynamic bone disease

In the 1980s the term “aplastic” or “adynamic” bone disease was introduced (Malluche and Faugere, 1986). Aluminum intoxication was previously the most frequent cause of low-turnover bone disease (aluminum-induced osteomalacia) since aluminum decreases the activation of OB and OC and causes an important defective mineralization (Malluche and Faugere, 1986; Nebeker and Coburn, 1986). ABD is characterized by suppressed bone formation, low cellularity, and thin osteoid seams, the last-mentioned being the most important difference with osteomalacia (wide osteoid volume) since mineralization is normal in ABD (Parfitt et al., 1987; Moe et al., 2006; Nagy et al., 2022). Minimodeling has been shown to potentially contribute to bone formation in dialysis patients with ABD, in the absence of remodelling stimulated by PTH, and this is especially the case in young patients with positive activities of daily living (Ubara et al., 2005). Minimodeling refers to the formation of bone by the action of OBs without prior resorption by OCs. Bone modeling can be divided into macromodeling or minimodeling depending on whether it is developed in cortical or cancellous bone, respectively. This process, which is very important during fetal and neonatal life, generates convex bone formation on smooth cement lines which do not express tartrate-resistant acid phosphatase, the hallmark of OC activity when bone formation is preceded by resorption. The first to use this term was Frost (Frost, 1966), and it was postulated later that minimodeling may continue in trabeculae throughout life (Frost, 1990). It seems to contribute to a significant percentage of bone volume in special conditions such as CKD, parathyroidectomized and/or patients with ABD, and it has been popularized with the advent of new dual medications against OP, such as romosozumab (Yamamoto et al., 2021). Increased mineralization of the minimodelling surface at the endocortical surface has also been observed in dialysis patients with ABD treated with the non-calcium-based phosphate binder lanthanum carbonate (Yajima et al., 2013).

The prevalence of ABD used to be much lower than that of osteitis fibrosa, but this pattern of bone damage has increased significantly, and in most recent studies it is described as the most prevalent (Frazão and Martins, 2009; Malluche et al., 2011; Nagy et al., 2022). It has even been proposed that ABD may be the predominant bone pattern in early stages of CKD (Massy and Drueke, 2017). The rising prevalence is probably due to patient’s increasing age and a higher prevalence of diabetes mellitus (relative hypoparathyroidism) (Jara et al., 1995; Brandenburg and Floege, 2008). In fact, however, the etiology of ABD is multifactorial and there are many other potential causes, including iatrogenic factors, malnutrition-inflammation syndrome, gonadal dysfunction, and the antagonistic effect of retained PTH fragments, among many others (Brandenburg and Floege, 2008; Bover et al., 2014). Calcium overload (oral or contained in the dialysate bath) and an excessive use of antiparathyroid (vitamin D or calcimimetics) agents have also been closely associated with ABD due to excessive suppression or inadequate normalization of PTH levels in CKD (Bover et al., 2014). The combination of relatively low levels of PTH and APs currently represents the best clinical basis for assessment of potential ABD beyond the gold standard bone biopsy (Couttenye et al., 1996; Moore et al., 2009; Sprague et al., 2016b; Bover et al., 2018). In fact, multifactorial hyporesponsiveness to PTH is a well-documented consequence of CKD (Evenepoel et al., 2016; Bover et al., 2021c) and a certain degree of secondary hyperparathyroidism is beneficial in CKD patients, not only because of the positive PTH phosphaturic effect but also in order to maintain a normal bone formation rate (Ketteler et al., 2022; Torregrosa et al., 2022). ABD has also been associated with a higher mortality, occasionally attributed to an increased number of fractures and accelerated vascular calcification (Bover et al., 2014).

A crucial aspect of low remodelling is that it promotes longer secondary mineralization, which may lead to brittle bones. It should be known that the osteoid mineralization process is carried out in two stages: primary mineralization, over the course of days, during which 50%–79% of the maximum mineralization is reached, and afterwards secondary mineralization begins (Ruffoni et al., 2007; Bala et al., 2010). Secondary mineralization is a slow process and develops in the course of months, contributing to the maximum mineralization and to an increase in the quantity and size of the crystals (Boivin and Meunier, 2003). Secondary mineralization occurs inversely to bone turnover. Thus, the greater the turnover the shorter the time in which secondary mineralization develops, and the lower the turnover the longer the duration of secondary mineralization (Boivin et al., 2009). In addition, the suppression of bone turnover can cause micro fissures that are difficult to repair in the presence of a low bone formation rate (Ng et al., 2016; Dong et al., 2019). In this context, the 2009 KDIGO CKD-MBD guideline recommended a bone biopsy prior to antiresorptive therapy in patients with CKD G4 to G5D, low BMD, and/or fragility fractures (Kidney Disease: Improving Global Outcomes KDIGOCKD–MBD Work Group, 2009). The rationale was that low BMD may be due to CKD-MBD (e.g., high PTH) and that lowering PTH is a safer and more appropriate therapy than an antiresorptive (Kidney Disease: Improving Global Outcomes KDIGOCKD–MBD Work Group, 2009). Moreover, there was concern that bisphosphonates could induce ABD, although this hypothesis was based upon a single cross-sectional study (Amerling et al., 2010). In the intervening period, studies in patients with CKD have not definitely demonstrated that bisphosphonates are a direct cause of ABD (Kidney Disease: Improving Global Outcomes KDIGO CKD-MBD Update Work Group, 2017; Torregrosa et al., 2022). Suppression of bone turnover is inherent to bisphosphonates and most treated patients develop a low bone formation rate (Evenepoel et al., 2021a), yet this treatment prevents fractures (Kidney Disease: Improving Global Outcomes KDIGO CKD-MBD Update Work Group, 2017). Suppression of bone turnover by bisphosphonates occurs even in the absence of CKD and there is no evidence that the level of remodelling suppression in CKD is greater than that in non-CKD counterparts (Allen and Aref, 2017). In any case, the implications of drug-induced suppression of bone turnover for bone strength are intensely debated (Evenepoel et al., 2021a). As we mentioned previously, low PTH levels as a proxy of low-bone turnover in CKD patients have been associated with increased fracture risk (Coco and Rush, 2000; Nitta et al., 2004) but it remains a matter of debate whether low bone turnover *per se* or the disease causing low bone turnover accounts for the perceived ABD-induced risk of fracture or adverse outcomes (Evenepoel et al., 2021a; Nagy et al., 2022).

It is noteworthy that in ABD there is a state of imbalance between the low circulating levels of bone anabolic factors such as IGF-1 and the increased expression of inhibitors of bone turnover such as sclerostin and Dkk1. This imbalance favors suppression of bone formation through inhibition of the Wnt-catenin pathway (Tanaka et al., 2015; Massy and Drueke, 2017). New treatments for OP with a dual action (anti-sclerostin antibodies with anabolic and antiresorptive properties) were shown to be promising in a rat model of progressive ROD (Moe et al., 2015)**.** It is noteworthy that these authors found efficacy in improving rat bone properties only when the PTH levels were low, also preventing calcium-induced vascular calcification, while no significant effect was observed in animals with high PTH levels.

## Osteoporosis

OP is defined as a systemic skeletal disease characterized by low bone mass and microarchitectural deterioration of bone tissue, leading to an increase in bone fragility and therefore a higher susceptibility to bone fractures (Compston et al., 2019). Consequently, the definition of OP includes not only bone *quantity* (mass) but also bone *quality* (microstructure), in addition to the important clinical outcome of fragility fractures (Compston et al., 2019). This concept was developed in 1993 by an International Consensus of experts, and the diagnostic criteria, which were also adopted by the WHO in 1994, use standard deviation (SD) scores of BMD in relation to the peak bone mass reached by young healthy women (WHO, 1994).

Briefly, in postmenopausal women, *OP* was defined as a BMD T-score lower than −2.5 SD below the average of young healthy women, and *osteopenia* as a BMD T-score between −1 and −2.5 SD below this average value (WHO, 1994). Thus, there is a significant relationship between BMD and fragility fractures, with a 1.5 to 2.6-fold increase in fracture risk for every SD decrease in BMD (Siris et al., 2004). Importantly, the diagnostic criteria recognize the importance of BMD in the pathogenesis of fragility fractures and also provide a tool to quantify the prevalence of OP (bone densitometry) (Compston et al., 2019). However, the utility of BMD as the sole clinical indicator for OP is limited since BMD is only one of multiple risk factors for fracture development. Actually, the majority of fragility fractures occur in individuals with BMD values *above* this threshold (less negative) (Siris et al., 2004; Compston et al., 2019). Accordingly, the National Bone Health Alliance Working Group published a position statement in 2014 which included not only BMD but also the presence of fragility fractures and the fracture risk assessed by the Fracture Risk Assessment Tool (FRAX) for the clinical diagnosis of OP (Siris et al., 2014). Apart from the BMD criteria, these experts also defined OP as the presence of a hip fragility fracture (with or without BMD), a non-hip fragility fracture (including vertebral, proximal humeral, pelvic, and distal forearm fractures) plus densitometric osteopenia, and a high fracture risk just based on a nationally-adapted FRAX score (Siris et al., 2014). Since bone quality is probably another important aspect to be taken into account which may be additionally affected in the presence of CKD (Tasnim et al., 2021), new tools are being developed to improve its assessment and improve fracture risk prediction both in the general population and in CKD patients (Bover et al., 2021a).

### Osteoporosis and chronic kidney disease

Studies assessing the presence of densitometric OP in patients with CKD are scarce since BMD assessment was not recommended in the previous 2009 KDIGO CKD-MBD guidelines (evidence 2B) (Kidney Disease: Improving Global Outcomes KDIGOCKD–MBD Work Group, 2009). However, as mentioned previously, an important shift occurred in the 2017 KDIGO CKD-MBD guidelines in the opposite direction (evidence 2B). Thus, it was now suggested that BMD testing could be used to assess fracture risk in patients with CKD G3a-G5D if results would impact treatment decisions (Kidney Disease: Improving Global Outcomes KDIGO CKD-MBD Update Work Group, 2017); the rationale being that new evidence had appeared, demonstrating that DXA does predict fractures also in patients with CKD (Kidney Disease: Improving Global Outcomes KDIGO CKD-MBD Update Work Group, 2017; Kidney Disease: Improving Global Outcomes KDIGOCKD–MBD Work Group, 2009; Pimentel et al., 2021)**.** The same suggestion has since been adopted in other national guidelines (Torregrosa et al., 2022). In fact, at least 4 prospective cohort studies using dual-energy X-ray absorptiometry (DXA) BMD and incident fractures demonstrated that DXA BMD predicted incident fractures across the spectrum from CKD G3a to G5D (Kidney Disease: Improving Global Outcomes KDIGO CKD-MBD Update Work Group, 2017). Nevertheless, it is important to recognize that DXA does not distinguish among different forms of ROD and it essentially evaluates *quantity* as opposed to *quality* of bone (Kidney Disease: Improving Global Outcomes KDIGO CKD-MBD Update Work Group, 2017; Pimentel et al., 2021). Consequently, BMD does not offer information either on bone microarchitecture [the trabecular bone score (TBS) may provide some additional clues] (Silva et al., 2014; Yun et al., 2020), bone turnover or mineralization, and cannot differentiate between OP, ABD, osteomalacia or osteitis fibrosa (Ginsberg and Ix, 2022). However, as mentioned before, BMD does predict fractures and may support decision-making together with bone biomarkers (Jørgensen et al., 2022a), even in the absence of a bone biopsy, according to recent guidelines which also draw attention to the associated vital risk (Kidney Disease: Improving Global Outcomes KDIGO CKD-MBD Update Work Group, 2017; Torregrosa et al., 2022).

Bone metabolism in CKD patients differs from that of the general population, depending on CKD stage, and type of kidney replacement therapy (i.e., hemodialysis, peritoneal dialysis, or kidney transplantation), among many other pathophysiological factors including bone location and histologic structure (Chen et al., 2014). Thus, cross-sectional analysis showed a significantly lower BMD at femoral neck and total hip and a significant higher serum PTH along with CKD stages (Cailleaux et al., 2021). Baseline age, gender, low body mass index, tobacco, and high PTH levels were significantly associated with low BMD (Cailleaux et al., 2021). Interestingly, the longitudinal bone loss observed in patients with CKD during the mean 4.3-year follow-up revealed a significant bone loss at the radius only, whereas BMD changes at the femoral neck were not associated with CKD stages or basal PTH levels (Cailleaux et al., 2021). These data invite to a better definition of the skeletal site and the monitoring schedule of serial BMD measurements in patients with CKD, and to investigate the changes of BMD and microarchitecture with high-resolution techniques, which may broaden the understanding and differential role of PTH on trabecular and cortical bone, especially in CKD patients (Cailleaux et al., 2021).

Different drugs in patients with this clinical condition (including glucocorticoids) may also affect bone metabolism, and it has to be taken into account that extensive vascular calcification or the presence of an arteriovenous fistula may additionally affect the diagnosis of OP (Evenepoel et al., 2021a; Pimentel et al., 2021). Moreover, drugs approved for OP are not simply the subject of theoretical concerns (ABD); rather some restrictions on use may be stipulated in their summary of product characteristics (SmPC) when decreased renal function is present (an example being bisphosphonates). However, growing experience with OP medications in patients with CKD has increased the confidence in using antiresorptive therapy in patients with low BMD and a high risk of fracture (Kidney Disease: Improving Global Outcomes KDIGO CKD-MBD Update Work Group, 2017; Torregrosa et al., 2022).

### Chronic kidney disease and fractures

It seems clear that patients with CKD sustain more fragility fractures than the general population (Pimentel et al., 2021), and the risk of fragility fracture has been reported to be up to 5 times higher in individuals with an estimated glomerular filtration rate (eGFR) of less than 15 ml/min/1.73 m^2^ compared to those in whom the eGFR exceeds 60 ml/min/1.73 m^2^ (Naylor et al., 2015; Pimentel et al., 2021). Additionally, it should be noted that the worse the CKD stage, the higher the fracture risk (Naylor et al., 2015). For example, the Canadian Multicentre Osteoporosis Study (CaMos) reported a fracture incidence ranging from 15.0/1,000 person-years in CKD G1 up to 46.3/1,000 person-years in CKD G4 (Naylor et al., 2015). Many other factors are involved in the increased fracture risk associated (or not) with CKD such as age, gender (the risk is higher in women), history of prior hip fracture, urine albumin levels, low body mass index, long dialysis vintage, and/or high and low-turnover bone disease (Pimentel et al., 2021). On the other hand, studies assessing the prevalence of vertebral fractures among individuals with CKD are scarce and clearly seem to underestimate such fractures, as the published rates have ranged between 1% and 20% (Pimentel et al., 2021). Furthermore, it should be underlined that there is a higher mortality risk after a fragility fracture (hip and non-hip fractures) in patients with CKD G4-G5 as compared to controls with an eGFR over 60 ml/min/1.73 m^2^ (de Bruin et al., 2020). Like many other authors, we have shown in a recent study that the presence of vertebral fractures is correlated with poorer survival and that these fractures are independent predictors of all-cause mortality (Castro-Alonso et al., 2020). Consequently, current nephrology guidelines have removed the requirement for bone biopsy prior to the use of antiresorptive drugs for OP because their use must be individualized in patients with CKD (Kidney Disease: Improving Global Outcomes KDIGO CKD-MBD Update Work Group, 2017; Torregrosa et al., 2022; Ketteler et al., 2017), and the risk/benefit ratio may be favourable also in these patients (Bover et al., 2019; Casado and Neyro, 2021; Torregrosa et al., 2022). Actually, it has been proposed that it is “time for action” even in the absence of randomized clinical trials (Moe and Nickolas, 2016), although it is still prudent to use these drugs with caution (Kidney Disease: Improving Global Outcomes KDIGO CKD-MBD Update Work Group, 2017; Torregrosa et al., 2022; Bover et al., 2019; Casado and Neyro, 2021).

## New bone metabolic pathways-new treatments

In recent years, we have learned more about the role of certain cell signalling pathways in the regulation of bone metabolism, examples being the OPG/RANKL (LGR4) system and the canonical Wnt-ß-catenin pathway. It is important that knowledge on these two pathophysiological pathways is further expanded, given that the latest approved antiosteoporotic treatments aim to modulate these signalling routes. Denosumab is an antibody against RANKL and romosozumab is an antibody against sclerostin. They are not specifically contraindicated in renal failure and therefore they may become a real therapeutic target for OP even in patients with CKD, also bearing the absence of chronic accumulation (Festuccia et al., 2017; Miller et al., 2022; Suzuki et al., 2022).

### Wnt-β-catenin signaling

**FIGURE 3:**
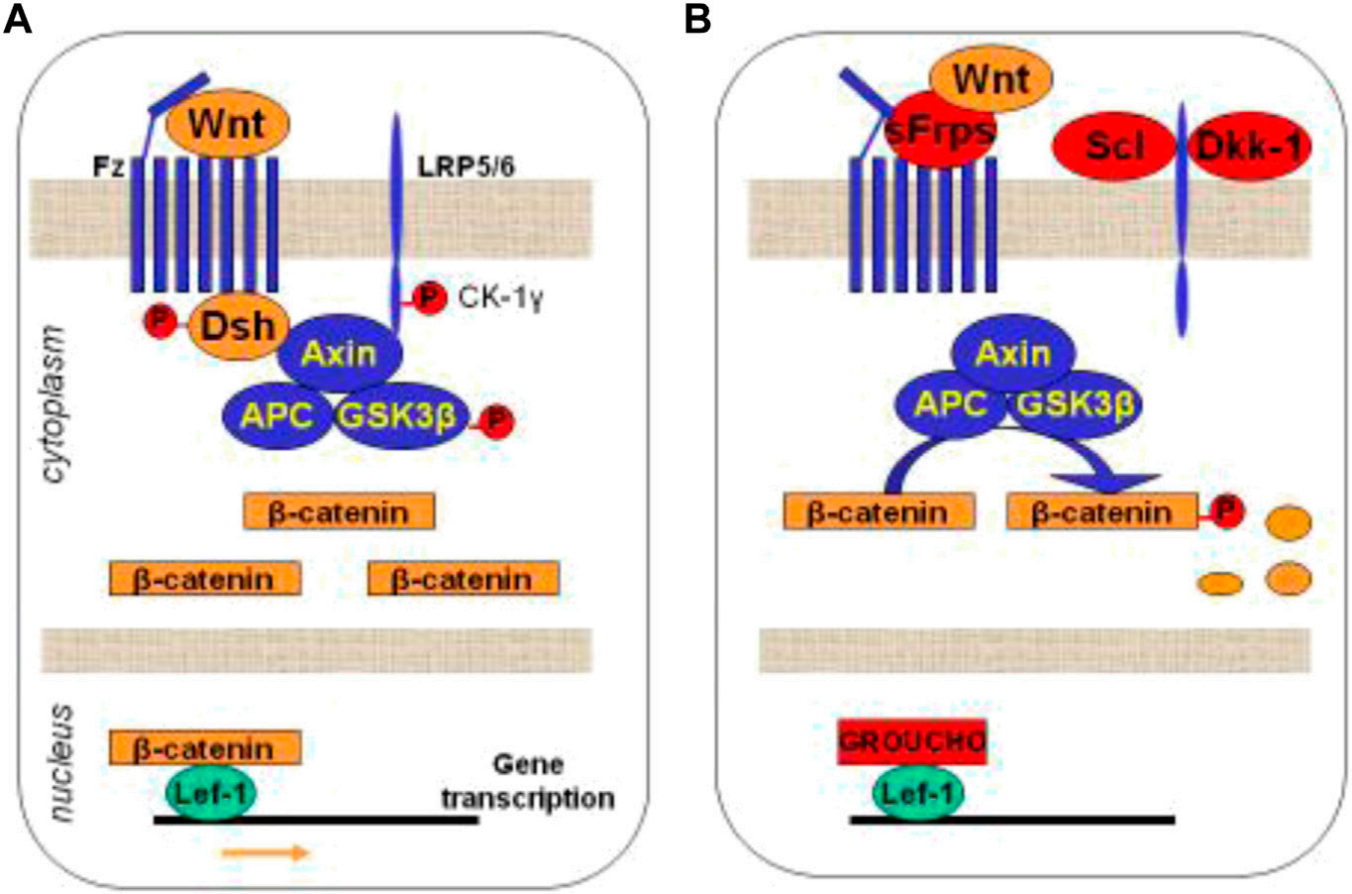
Schematic representation of the Wnt-ß-catenin signalling pathway. **(A)** Activation of the intracellular cascade depending on the union between Wnt protein and its receptors. **(B)** Inhibition of Wnt signaling by its antagonists, blocking the union between Wnt protein and its receptors. Wnt: Wnt protein; Fz: frizzled; LRP5: low-density lipoprotein receptor (LDLR)-related protein 5 or LRP6; GSK-3b: glycogen synthetase kinase 3; APC: tumor suppressor adenomatous polyposis coli; Lef-1: Tcf/lymphoid enhancer-binding factor; Sfrp: secreted Fz-related-proteins; Scl: Sclerostin; Dkk1: dickkopf–related protein 1. Adapted from [Guañabens N, Curr Osteoporos Rep 2014].

We have already mentioned that the Wnt-β-catenin is an intracellular signaling pathway that has emerged as a key regulator of osteoblastogenesis (Gordon and Nusse, 2006). Its regulation occurs mainly through its antagonists, sclerostin and Dkk-1, which are primarily expressed by osteocytes (Gordon and Nusse, 2006). In brief, when the Wnt protein binds to the dual receptor complex, which comprises frizzled (Fz) and either low-density lipoprotein receptor (LDLR)-related protein 5 (LRP5) or LRP6, ß-catenin is accumulated in the cytoplasm and enters the nucleus, where it is associated with a transcription factor, regulating the expression of canonical Wnt target genes such as *WISP1* and *RUNX*2 (Gordon and Nusse, 2006) (Figure 3). Consequently, the activation of Wnt signalling finally induces the differentiation of OB precursors towards mature OBs and prevents OB (and osteocyte) apoptosis, resulting in increases in bone mass. However, when sclerostin or Dkk-1 binds to the Wnt receptors (LRP-5/6 membrane), they prevent the binding of the Wnt protein to its receptors, and the cytoplasmatic ß-catenin protein is phosphorylated and degraded. Therefore, ß-catenin cannot enter the nucleus and cannot activate osteoblastogenesis. Other inhibitors, such as the Sfrp 1and 4, (upregulated in CKD) do not specifically bind to LRPs but compete with the Wnt ligand for the binding to the receptor (Gordon and Nusse, 2006). As a consequence, the inhibition of Wnt signalling (by the absence of Wnt protein or the presence of antagonists) leads to downregulation of bone formation, probably leading to a lower bone mass (Gordon and Nusse, 2006; Baron and Kneissel, 2013b). In addition, Wnt-ß-catenin signalling is critical not only for osteoblastogenesis but also for the regulation of osteoclastogenesis (Wijenayaka et al., 2011) through the RANKL/OPG system (Figure 3).

### Sclerostin

Sclerostin is a 22-kDa protein that is a member of the cystatin knot family of proteins and the product of the *SOST* gene (Brandenburg et al., 2019). Loss of function mutations of the *SOST* gene have been reported in van Buchem disease (sclerosteosis; a hereditary sclerosing bone dysplasia) (), and this represented the starting point for development of an anti-sclerostin antibody which has recently been approved for the treatment of OP (Baron and Kneissel, 2013b). As mentioned above, sclerostin downregulates osteoblastogenesis but also promotes osteoclastogenesis through activation of the RANKL/OPG system; thus the antibody offers a unique dual positive action (Wijenayaka et al., 2011). Sclerostin is also expressed by osteoclast precursors, hepatocytes, and renal and vascular cells, but little is known about the systemic effects of this molecule [186].

In postmenopausal women, increased circulating sclerostin values have been associated with OP and an increased risk of fragility fractures (Clarke and Drake, 2013). It has also been described an increase of circulating sclerostin values in some clinical conditions associated with OP such as type 2 diabetes mellitus, glucocorticoid treatment, multiple myeloma or CKD; whereas decreased values ​​of sclerostin have been described in patients with hyperparathyroidism and under osteoanabolic treatment with teriparatide (Moysés and Schiavi, 2015).

### Sclerostin in chronic kidney disease

In CKD, circulating levels of sclerostin increase as kidney function declines (Moysés and Schiavi, 2015; Massy and Drueke, 2017; Figurek et al., 2020). Thus, the level has been reported to be 5 times higher in patients with CKD G5D (Figurek et al., 2020). Additionally, increased sclerostin values have been described in individuals undergoing hemodialysis (Moysés and Schiavi, 2015). Curiously, it seems that peritoneal dialysis decreases sclerostin circulating levels (as 2.5 times higher values have been found in the dialysate compared to urine), whereas after a conventional hemodialysis session the circulating levels of sclerostin remain unaltered (Figurek et al., 2020). In this context, a small prospective study has shown suppression of the increase in sclerostin level in hemodialysis using a medium cut-off dialysis filter (Ahn et al., 2021).

The pathophysiology of sclerostin in CKD and its consequences are not clear yet. On the one side, sclerostin is overexpressed by osteocytes and by injured kidney cells (Moysés and Schiavi, 2015), while on the other hand there is an increased tubular excretion of sclerostin with a reduction in renal function (Moysés and Schiavi, 2015; Figurek et al., 2020). It is well known that after kidney transplantation serum sclerostin values are rapidly restored to the normal range (Moysés and Schiavi, 2015). Altogether suggests that a sclerostin accumulation occurs in CKD, and it has been reported that it may be present even at early CKD stages in an attempt to prevent bone loss (Massy and Drueke, 2017) as a result of both a multifactorial increase in expression and a decrease in elimination (Moysés and Schiavi, 2015)**.** Moreover, it seems that sclerostin also intervenes in the relationship among phosphate, FGF23 and bone in CKD. Thus, a positive correlation has been described between sclerostin, serum phosphate, and FGF23, while, conversely, a negative relationship with PTH has been reported (Moysés and Schiavi, 2015; Figurek et al., 2020)**.** Additionally, high sclerostin levels have been associated with PTH resistance or hyporesponsiveness to PTH in CKD (Massy and Drueke, 2017; Figurek et al., 2020; Bover et al., 2021c). Other molecules beyond PTH and phosphate (and probably FGF23) also seem to regulate the expression of sclerostin, such as calcitriol, BMPs, TNF, and prednisone (Moysés and Schiavi, 2015)**,** suppressing sclerostin expression from osteocytes, and increasing the rate of bone remodelling (Figurek et al., 2020).

Finally, an inverse relationship has been described between sclerostin and bone AP (Figurek et al., 2020). A recent study assessed the relationship between bone histomorphometric parameters from patients with CKD G3-G4 and circulating levels of the Wnt antagonists sclerostin and Dkk-1 (Neto et al., 2021). It was observed that individuals with low-turnover bone disease (diagnosed by bone biopsy) had higher circulating sclerostin levels and lower DKK-1 and RANKL levels as compared to individuals with high-turnover bone disease or normal bone histology (Neto et al., 2021), demonstrating an association between higher circulating sclerostin values and lower bone remodelling (low bone turnover). Additionally, in a CKD animal model of ABD, high dietary phosphate intake was associated with high osteocyte *SOST* expression (Antoine et al., 2020). While more studies are needed to assess the usefulness of the circulating values of sclerostin as a clinical tool to identify patients with low-turnover bone disease in CKD, taking into account all the data it may be concluded that blocking sclerostin could be an interesting treatment target for those patients with low-bone turnover or at least some CKD patients with OP due its dual positive action (Antoine et al., 2020). Romosozumab has recently been approved for the treatment of postmenopausal OP, although in Europe it carries a black box warning that it should not be initiated in patients with myocardial infarction or stroke in the preceding year (Anthony, 2019). Initial positive experiences in CKD patients are being currently published (Sato et al., 2021; Miller et al., 2022; Suzuki et al., 2022).

## Denosumab and romosozumab

We have already briefly discussed both the OPG-RANKL-RANK system and the Wnt-β-catenin signaling pathway, and that these monoclonal antibodies (anti-RANKL and antisclerostin, respectively) are not contraindicated in CKD or dialysis patients with OP. Nevertheless, their use in CKD may be associated with specific problems (at least in advanced stages), which are described elsewhere (Festuccia et al., 2017; Bover et al., 2019; Miller et al., 2022; Suzuki et al., 2022)”. Briefly, hypocalcemia is more frequently observed in CKD patients and therefore an adequate repletion of calcium and vitamin D is recommended (Miller et al., 2022), especially in dialysis patients. Most importantly, the favorable skeletal effects of these monoclonal antibodies may reverse quickly upon discontinuation, especially after denosumab withdrawal, because of a vast increase of OC number and activity, which may lead to a subsequent profound increase of bone turnover to pre-treatment or even above pre-treatment values, a phenomenon commonly described as “rebound phenomenon” (Casado and Neyro, 2021).

In the case of denosumab, subsequent multiple vertebral fractures have been described upon discontinuation (Tsourdi et al., 2017; McClung et al., 2020). Risk was similar to that observed in the placebo group in the randomized clinical trials (Kim et al., 2022). A recent new hypothesis for the rebound effect after denosumab cessation is by the phenomenon to OB recicling from osteomorphs, a newly described cell state (Kim et al., 2022). Consequently, in case that a cessation of treatment is deemed necessary either by patient’s decision, or medical reasons such as low adherence, or after reaching a determined T-score at BMD, or after completing the 12-month treatment (in the case of romosozumab), guidelines recommend different approaches and/or sequential treatments which are more detailed and specific after denosumab suppression (Tsourdi et al., 2017; McClung et al., 2020; Kendler et al., 2022; Kim et al., 2022).

In summary, CKD is a highly prevalent condition and recent years have witnessed important advances in the understanding of the associated mineral metabolism disorders. The *systemic* complex CKD-MBD has already been universally accepted because of its clear association with cardiovascular disease and extremely high mortality rates. CKD-MBD includes a bone component which is no longer just represented by the classical ROD or disorders of bone remodelling. In fact, it is now very well known that CKD is associated with a higher risk of falls and fractures, and higher mortality rates when a fracture occurs. New guidelines suggest that BMD should also be assessed in CKD patients if results will impact clinical decisions (i.e., individualized prescription of anti-OP drugs). Several illustrative algorithms for CKD patients have already been published, broadly calling for a shift from nihilism to pragmatism (Pimentel et al., 2017; Evenepoel et al., 2021b; Evenepoel et al., 2021c; Pimentel et al., 2021; Casado et al., 2022; Ginsberg and Ix, 2022; Haarhaus et al., 2022). However, it is still prudent to use these drugs (bisphosphonates, denosumab, recombinant PTH and romosozumab) with caution, especially in advanced kidney disease, balancing the risk/benefit ratio, since, as documented in this article, pathophysiological pathways are extremely intricate and not completely unraveled yet.

## References

[B1] Abou-Samra A-B. Juppner H. Force T. Freeman M. W. Kong X-F. Schipani E. (1992). Expression cloning of a common receptor for parathyroid hormone and parathyroid hormone-related peptide from rat osteoblast-like cells: A single receptor stimulates intracellular accumulation of both cAMP and inositol trisphosphates and increases intracellular free calcium. Proc. Natl. 5. Acad. Sci. U. S. A. 89, 2732–2736. 10.1073/pnas.89.7.2732 PMC48736 1313566

[B2] AgapovaFangSugatani O. A. Y. T. Seifert M. E. Hruska K. A. (2016). Ligand trap for the activin type IIA receptor protects against vascular disease and renal fibrosis in mice with chronic kidney disease. Kidney Int. 89, 1231–1243. 10.1016/j.kint.2016.02.002 27165838 PMC4868771

[B3] Ahn S. H. Ko M. M. Song J. H. Jung J. H. (2021). Changes in plasma sclerostin level associated with use of a medium cut-off dialyzer in end-stage renal disease. Kidney Res. Clin. Pract. 40 (1), 120–134. 10.23876/j.krcp.20.173 33745263 PMC8041631

[B4] Alberto Ortiz (2022). Ckd: The burden of disease invisible to research funders. Nefrología 42 (1), 65–84. 10.1016/j.nefroe.2021.09.005 PMC9040466 36153901

[B5] Albright F. Reifenstein E. C. (1948). The parathyroid glands and metabolic bone disease. Selected studies. Baltimore: Williams & Wilkins Co.

[B6] Allan E. H. Häusler K. D. Wei T. Gooi J. H. Quinn J. M. W. Crimeen-Irwin B. (2008). EphrinB2 regulation by PTH and PTHrP revealed by molecular profiling in differentiating osteoblasts. J. Bone Min. Res. 23 (8), 1170–1181. 10.1359/jbmr.080324 18627264

[B7] Allen M. R. Aref M. W. (2017). What animal models have taught us about the safety and efficacy of bisphosphonates in chronic kidney disease. Curr. Osteoporos. Rep. 15 (3), 171–177. PMID: 28432595; PMCID: PMC9055792. 10.1007/s11914-017-0361-4 28432595 PMC9055792

[B8] Amerling R. Harbord N. B. James P. Feinfeld D. A. (2010). Bisphosphonate use in chronic kidney disease: Association with adynamic bone disease in a bone histology series. Blood Purif. 29, 293–299. 10.1159/000276666 20090316

[B9] Anthony M. (2019). Romosozumab: First global approval. Drugs 79, 471–476. 10.1007/s40265-019-01072-6 30805895

[B10] Antoine B. Evenepoel P. Paquot F. Malaisec O. Cavalier E. Delanaye P. (2020). Sclerostin within the chronic kidney disease spectrum. Clin. Chim. Acta 502, 84–90. 10.1016/j.cca.2019.12.008 31866333

[B11] Arumugam B. Vishal M. Shreya S. Malavika D. Rajpriya V. He Z. (2019). Parathyroid hormone-stimulation of Runx2 during osteoblast differentiation via the regulation of lnc-SUPT3H-1:16 (RUNX2-AS1:32) and miR-6797-5p. Biochimie 158, 43–52. Epub 2018 Dec 15. 10.1016/j.biochi.2018.12.006 30562548

[B12] Bala Y. Farlay D. Delmas P. D. Meunier P. J. Boivin G. (2010). Time sequence of secondary mineralization and microhardness in cortical and cancellous bone from ewes. Bone 46 (4), 1204–1212. Epub 2009 Dec 5. PMID: 19969115. 10.1016/j.bone.2009.11.032 19969115

[B13] Baron R. Kneissel M. (2013). WNT signaling in bone homeostasis and disease: From human mutations to treatments. Nat. Med. 19 (2), 179–192. Epub 2013 Feb 6. PMID: 23389618. 10.1038/nm.3074 23389618

[B14] Baron R. Kneissel M. (2013). WNT signaling in bone homeostasis and disease: From human mutations to treatments. Nat. Med. 19 (2), 179–192. 10.1038/nm.3074 23389618

[B15] Bellido T. Ali A. A. Gubrij I. Plotkin L. I. Fu Q. O’Brien C. A. (2005). Chronic elevation of parathyroid hormone in mice reduces expression of sclerostin by osteocytes: A novel mechanism for hormonal control of osteoblastogenesis. Endocrinology 146 (11), 4577–4583. 10.1210/en.2005-0239 16081646

[B16] Ben-awadh A. N. Delgado-Calle J. Tu X. Kuhlenschmidt K. Allen M. R. Plotkin L. I. (2014). Parathyroid hormone receptor signaling induces bone resorption in the adult skeleton by directly regulating the RANKL gene in osteocytes. Endocrinology 155 (8), 2797–2809. 10.1210/en.2014-1046 24877630 PMC4098003

[B17] Bikle D. D. Sakata T. Leary C. Elalieh H. Ginzinger D. Rosen C. J. (2002). Insulin-like growth factor I is required for the anabolic actions of parathyroid hormone on mouse bone. J. Bone Min. Res. [Internet] 17 (9), 1570–1578. 10.1359/jbmr.2002.17.9.1570 12211426

[B18] Boivin G. Farlay D. Bala Y. Doublier A. Meunier P. J. Delmas P. D. (2009). Influence of remodeling on the mineralization of bone tissue. Osteoporos. Int. 20, 1023–1026. 10.1007/s00198-009-0861-x 19340504 PMC2904474

[B19] Boivin G. Meunier P. J. (2003). Methodological considerations in measurement of bone mineral content. Osteoporos. Int. 14 (5), 22–S27. 10.1007/s00198-003-1469-1 14504702

[B20] Bonewald L. F. Johnson M. L. (2008). Osteocytes, mechanosensing and Wnt signaling. Bone 42 (4), 606–615. 10.1016/j.bone.2007.12.224 18280232 PMC2349095

[B21] Bonewald L. F. (2007). Osteocytes as dynamic multifunctional cells. Ann. N. Y. Acad. Sci. 1116 (1), 281–290. 10.1196/annals.1402.018 17646259

[B22] Bover J. Ureña -Torres P. Laiz Alonso A. M. Torregrosa J. V. Rodríguez-García M. Castro-Alonso C. (2019). Osteoporosis, bone mineral density and CKD-MBD (II): Therapeutic implications. Nefrologia 39, 227–242. 10.1016/j.nefro.2018.10.009 30797619

[B23] Bover J. Ureña P. Brandenburg V. Goldsmith D. Ruiz C. DaSilva I. (2014). Adynamic bone disease: From bone to vessels in chronic kidney disease. Semin. Nephrol. 34 (6), 626–640. PMID: 25498381. 10.1016/j.semnephrol.2014.09.008 25498381

[B24] Bover J. Ureña-Torres P. Cozzolino M. Rodríguez-García M. Gómez-Alonso C. (2021). The non-invasive diagnosis of bone; disorders in CKD. Calcif. Tissue Int. 108, 512–527. 10.1007/s00223-020-00781-5 33398414

[B25] Bover J. Aguilar A. Arana C. Molina P. Jackson O. Berna G. (2021). Clinical approach to vascular calcification in patients with non-dialysis dependent chronic kidney disease: Mineral-bone disorder-related aspects. Front. Med. 8, 642718. 10.3389/fmed.2021.642718 PMC8171667 34095165

[B26] Bover J. Arana C. Ureña P. Torres A. Martín-Malo A. Fayos L. (2021). Hyporesponsiveness or resistance to the action of parathyroid hormone in chronic kidney disease. Nefrología. 41, 514–528. 10.1016/j.nefroe.2021.11.014 36165134

[B27] Bover J. Ureña P. Aguilar A. Mazzaferro S. Benito S. López-Báez V. (2018). Alkaline phosphatases in the complex chronic kidney disease-mineral and bone disorders. Calcif. Tissue Int. 103 (2), 111–124. 10.1007/s00223-018-0399-z 29445837

[B28] Brandenburg V. M. Floege J. (2008). Adynamic bone disease-bone and beyond. NDT Plus 1 (3), 135–147. 10.1093/ndtplus/sfn040 25983860 PMC4421169

[B29] Brandenburg V. M. Verhulst A. Babler A. D’Haese P. C. Evenepoel P. Kaesler N. (2019). Sclerostin in chronic kidney disease–mineral bone disorder think first before you block it. Nephrol. Dial. Transpl. 34, 408–414. 10.1093/ndt/gfy129 29846712

[B30] Brewer H. B. Jr Ronan R. (1970). Bovine parathyroid hormone: Amino acid sequence. Proc. Natl. Acad. Sci. U. S. A. 67 (4), 1862–1869. 10.1073/pnas.67.4.1862 5275384 PMC283440

[B31] Burger E. H. Klein-Nulend J. Smit T. H. (2003). Strain-derived canalicular fluid flow regulates osteoclast activity in a remodelling osteon- a proposal. J. Biomech. 36 (10), 1453–1459. 10.1016/s0021-9290(03)00126-x 14499294

[B32] Cailleaux P-E. Ostertag A. Metzger M. Stengel B. Boucquemont J. Houillier P. (2021). Longitudinal bone loss occurs at the radius in CKD. Kidney Int. Rep. 6 (6), 1525–1536. 10.1016/j.ekir.2021.03.874 34169193 PMC8207310

[B33] Cameron D. A. Paschall H. A. Robinson R. A. (1967). Changes in the fine structure of bone cells after the administration of parathyroid extract. J. Cell Biol. 33 (1), 1–14. 10.1083/jcb.33.1.1 6033936 PMC2107294

[B34] Carrillo-López N. Panizo S. Alonso-Montes C. Román-García P. Rodríguez I. Martínez-Salgado C. (2016). Direct inhibition of osteoblastic Wnt pathway by fibroblast growth factor 23 contributes to bone loss in chronic kidney disease. Kidney Int. 90 (1), 77–89. 10.1016/j.kint.2016.01.024 27165819

[B35] Casado E. Bover J. Gómez-Alonso C. Navarro-González J. F. (2022). Osteoporosis in chronic kidney disease: A essential challenge. Med. Clin. Barc. 158 (1), 27–34. Epub 2021 Jun 18. PMID: 34154811. 10.1016/j.medcli.2021.05.007 34154811

[B36] Casado E. Neyro J. L. (2021). Tratamiento secuencial en osteoporosis. Nuevas tendencias. Rev. Osteoporos. Metab. Min. 13 (4), 107–116. 10.4321/s1889-836x2021000400002

[B37] Castro-Alonso C. D'Marco L. Pomes J. Del Amo Conill M. García-Diez A. I. Molina P. (2020). Prevalence of vertebral fractures and their prognostic significance in the survival in patients with chronic kidney disease stages 3‒5 not on dialysis. J. Clin. Med. 9 (5), 1604. 10.3390/jcm9051604 32466297 PMC7291319

[B38] Cejka D. Jager-Lansky A. Kieweg H. Weber M. Bieglmayer C. Haider D. G. (2012). Sclerostin serum levels correlate positively with bone mineral density and microarchitecture in haemodialysis patients. Nephrol. Dial. Trans. plant. 27 (1), 226–230. 10.1093/ndt/gfr270 21613383

[B39] Cejka D. Kodras K. Bader T. Haas M. (2010). Treatment of hemodialysis-associated adynamic bone disease with teriparatide (PTH1-34): A pilot study. Kidney Blood Press Res. 33 (3), 221–226. Epub 2010 Jun 24. PMID: 20588059. 10.1159/000316708 20588059

[B40] Chen Y. J. Kung P. T. Wang Y. H. Huang C. C. Hsu S. C. Tsai W. C. (2014). Greater risk of hip fracture in hemodialysis than in peritoneal dialysis. Osteoporos. Int. 25, 1513–1518. 10.1007/s00198-014-2632-6 24557014

[B41] Cianciolo G. La Manna G. Capelli I. Gasperoni L. Galassi A. Ciceri P. (2021). The role of activin: The other side of chronic kidney disease-mineral bone disorder? Nephrol. Dial. Transpl. 36 (6), 966–974. 10.1093/ndt/gfaa203 32940690

[B42] Clarke B. L. Drake M. T. (2013). Clinical utility of serum sclerostin measurements. Bonekey Rep. 2, 361. 10.1038/bonekey.2013.95 24578825 PMC3936109

[B43] Coco M. Rush H. (2000). Increased incidence of hip fractures in dialysis patients with low serum parathyroid hormone. Am. J. Kidney Dis. 36 (6), 1115–1121. 10.1053/ajkd.2000.19812 11096034

[B44] Compston J. E. McClung M. R. Leslie W. D. (2019). Osteoporos. Lancet 393, 364–376. 10.1016/S0140-6736(18)32112-3 30696576

[B45] Couttenye M. M. D’Haese P. C. Van Hoof V. O. Lemoniatou E. Goodman W. Verpooten G. A. (1996). Low serum levels of alkaline phosphatase of bone origin: A good marker of adynamic bone disease in haemodialysis patients. Nephrol. Dial. Transpl. 11 (6), 1065–1072. 10.1093/oxfordjournals.ndt.a027457 8671970

[B46] Crane J. L. Cao X. (2014). Function of matrix IGF-1 in coupling bone resorption and formation. J. Mol. Med. 92 (2), 107–115. 10.1007/s00109-013-1084-3 24068256 PMC3946714

[B47] Csaba P. (2022). Epidemiology of chronic kidney disease: An update 2022. Kidney Int. Suppl. 12, 7–11. 10.1016/j.kisu.2021.11.003 PMC9073222 35529086

[B48] Cunningham J. Locatelli F. Rodriguez M. (2011). Secondary hyperparathyroidism. Pathogenesis, disease progression, and therapeutic options. Clin. J. Am. Soc. Nephrol. 6 (4), 913–921. 10.2215/CJN.06040710 21454719

[B49] Day T. F. Guo X. Garrett-Beal L. Yang Y. (2005). Wnt/beta-catenin signaling in mesenchymal progenitors controls osteoblast and chondrocyte differentiation during vertebrate skeletogenesis. Dev. Cell 8 (5), 739–750. 10.1016/j.devcel.2005.03.016 15866164

[B50] de Bruin I. J. A. Wyers C. E. Souverein P. C. van Staa T. P. Geusens P. P. M. M. van den Bergh J. P. W. (2020). The risk of new fragility fractures in patients with chronic kidney disease and hip fracture-a population-based cohort study in the UK. Osteoporos. Int. 31 (8), 1487–1497. 10.1007/s00198-020-05351-x 32266436 PMC7360657

[B51] De Pasquale L. Gobatti D. Ravini M. L. Barassi A. Porreca W. Melzi d’Eril G. V. (2008). Intra-operative testing for parathyroid hormone: The central laboratory option. J. Endocrinol. Invest. 31 (1), 62–67. 10.1007/BF03345568 18296907

[B52] Dong B. Endo I. Ohnishi Y. Mitsui Y. Kurahashi K. Kanai M. (2019). Persistent activation of calcium-sensing receptor suppresses bone turnover, increases microcracks, and decreases bone strength: Increased microcracks with reduced bone strength in adh1 mice. JBMR Plus 3 (7), e10182. 10.1002/jbm4.10182 31372589 PMC6659446

[B53] Drake M. T. Srinivasan B. Modder U. I. Peterson J. M. McCready L. K. Lawrence Riggs B. (2010). Effects of parathyroid hormone treatment on circulating sclerostin levels in postmenopausal women. J. Clin. Endocrinol. Metab. Novemb. 95 (11), 5056–5062. 10.1210/jc.2010-0720 PMC2968729 20631014

[B54] Drüeke T. B. Massy Z. A. (2016). Changing bone patterns with progression of chronic kidney disease. Kidney Int. 89 (2), 289–302. PMID: 26806832. 10.1016/j.kint.2015.12.004 26806832

[B55] Elshahat S. Paul C. Maxwell A. P. Griffin M. O’Brien T. O’Neill C. (2020). The impact of chronic kidney disease on developed countries from a health economics perspective: A systematic scoping review. PLoS One 15 (3), e0230512. 10.1371/journal.pone.0230512 32208435 PMC7092970

[B56] Evenepoel P. Bover J. Ureña Torres P. (2016). Parathyroid hormone metabolism and signaling in health and chronic kidney disease. Kidney Int. 90, 1184–1190. 10.1016/j.kint.2016.06.041 27653840

[B57] Evenepoel P. Cunningham J. Ferrari S. Haarhaus M. Javaid M. K. Lafage-Proust M. H. (2021). European Consensus Statement on the diagnosis and management of osteoporosis in chronic kidney disease stages G4-G5D. Nephrol. Dial. Transpl. 36, 42–59. 10.1093/ndt/gfaa192 33098421

[B58] Evenepoel P. Cunningham J. Ferrari S. Haarhaus M. Javaid M. K. Lafage-Proust M. H. (2021). Diagnosis and management of osteoporosis in chronic kidney disease stages 4 to 5D: A call for a shift from nihilism to pragmatism. Osteoporos. Int. 32 (12), 2397–2405. Epub 2021 Jun 15. PMID: 34129059. 10.1007/s00198-021-05975-7 34129059

[B59] Evenepoel P. Cunningham J. Ferrari S. Haarhaus M. Javaid M. K. Lafage-Proust M. H. (2021). European Consensus Statement on the diagnosis and management of osteoporosis in chronic kidney disease stages G4-G5D. Nephrol. Dial. Transpl. 36 (1), 42–59. 10.1093/ndt/gfaa192 33098421

[B60] Evenepoel P. D’Haese P. Bacchetta J. Cannata-Andia J. Ferreira A. Haarhaus M. (2017). Bone biopsy practice patterns across Europe: The European renal osteodystrophy initiative-a position paper. Nephrol. Dial. Transpl. 32, 1608–1613. 10.1093/ndt/gfw468 28339949

[B61] Fei Y. Shimizu E. McBurney M. W. Partridge N. C. (2015). Sirtuin 1 is a negative regulator of parathyroid hormone stimulation of matrix metalloproteinase 13 expression in osteoblastic cells: Role of sirtuin 1 in the action of PTH on osteoblasts. J. Biol. Chem. 290 (13), 8373–8382. 10.1074/jbc.M114.602763 25631045 PMC4375490

[B62] Feng X. McDonald J. M. (2011). Disorders of bone remodeling. Annu. Rev. Pathol. 6, 121–145. PMID: 20936937; PMCID: PMC3571087. 10.1146/annurev-pathol-011110-130203 20936937 PMC3571087

[B63] Feng X. Teitelbaum S. L. (2013). Osteoclasts: New insights. Bone Res. 1 (1), 11–26. 10.4248/BR201301003 26273491 PMC4472093

[B64] Festuccia F. Jafari M. T. Moioli A. Fofi C. Barberi S. Amendola S. (2017). Safety and efficacy of denosumab in osteoporotic hemodialysed patients. J. Nephrol. 30, 271–279. 10.1007/s40620-016-0334-1 27394428

[B65] Figurek A. Rroji M. Spasovski G. (2020). Sclerostin: A new biomarker of CKD-MBD. Int. Urol. Nephrol. 52 (1), 107–113. Epub 2019 Oct 14. PMID: 31612420. 10.1007/s11255-019-02290-3 31612420

[B66] Filipowska J. Kondegowda N. G. Leon-Rivera N. Dhawan S. Vasavada R. C. (2022). LGR4, a G Protein-Coupled receptor with a systemic role: From development to metabolic regulation. Front. Endocrinol. (Lausanne) 13, 867001. 10.3389/fendo.2022.867001 35707461 PMC9190282

[B67] Frazão J. M. Martins P. (2009). Adynamic bone disease: Clinical and therapeutic implications. Curr. Opin. Nephrol. Hypertens. 18 (4), 303–307. PMID: 19424062. 10.1097/MNH.0b013e32832c4df0 19424062

[B68] Frost H. M. (1966). Bone dynamics in metabolic bone disease. J. Bone Jt. Surg. Am. 48, 1192–1203. 10.2106/00004623-196648060-00018 5331235

[B69] Frost H. M. (1990). Skeletal structural adaptations to mechanical usage (SATMU):1. Redefining wolff’s law: The bone modeling problem. Anat. Rec. 226, 403–413. 10.1002/ar.1092260402 2184695

[B70] Fu Q. Jilka R. L. Manolagas S. C. O’Brien C. A. (2002). Parathyroid hormone stimulates receptor activator of NFkappa B ligand and inhibits osteoprotegerin expression via protein kinase A activation of cAMP-response element-binding protein. J. Biol. Chem. 277 (50), 48868–48875. 10.1074/jbc.M208494200 12364326

[B71] Fu Q. Manolagas S. C. O’Brien C. A. (2006). Parathyroid hormone controls receptor activator of NF-kappaB ligand gene expression via a distant transcriptional enhancer. Mol. Cell Biol. 26 (17), 6453–6468. 10.1128/MCB.00356-06 16914731 PMC1592840

[B72] Ginsberg C. Ix J. H. (2022). Diagnosis and management of osteoporosis in advanced kidney disease: A review. Am. J. Kidney Dis. 79 (3), 427–436. Epub 2021 Aug 20. PMID: 34419519. 10.1053/j.ajkd.2021.06.031 34419519

[B73] Goltzman David (2018). Physiology of parathyroid hormone. Endocrinol. Metab. Clin. North Am. 47 (4), 743–758. Epub 2018 Oct 11. 10.1016/j.ecl.2018.07.003 30390810

[B74] Goltzman D. Mannstadt M. Marcocci C. (2018). “Physiology of the calcium-parathyroid hormone-vitamin D Axis,” in Vitamin D in clinical medicine. Editors Giustina A. Bilezikian J. P. (Front Horm Res. Basel, Karger), 1–13. 10.1159/000486060 29597231

[B75] Gordon M. D. Nusse R. (2006). Wnt signaling: Multiple pathways, multiple receptors, and múltiple transcription factors. J. Biol. Chem. 281 (32), 22429–22433. 10.1074/jbc.R600015200 16793760

[B76] Gori F. Hofbauer L. C. Dunstan C. R. Spelsberg T. C. Khosla S. Riggs B. L. (2000). The expression of osteoprotegerin and RANK ligand and the support of osteoclast formation by stromal-osteoblast lineage cells is developmentally regulated. Endocrinology 141 (12), 4768–4776. 10.1210/endo.141.12.7840 11108292

[B77] Guo J. Liu M. Yang D. Bouxsein M. L. Saito H. Galvin R. J. S. (2010). Suppression of wnt signaling by Dkk1 attenuates PTH-mediated stromal cell response and new bone formation. Cell Metab. 11 (2), 161–171. 10.1016/j.cmet.2009.12.007 20142103 PMC2819982

[B78] Gutiérrez O. M. Mannstadt M. Isakova T. Rauh-Hain J. A. Tamez H. Shah A. (2008). Fibroblast growth factor 23 and mortality among patients undergoing hemodialysis. N. Engl. J. Med. 359, 584–592. 10.1056/NEJMoa0706130 18687639 PMC2890264

[B79] Haarhaus M. Aaltonen L. Cejka D. Cozzolino M. de Jong R. T. D'Haese P. (2022). Management of fracture risk in CKD-traditional and novel approaches. Clin. Kidney J. 16 (3), 456–472. PMID: 36865010; PMCID: PMC9972845. 10.1093/ckj/sfac230 36865010 PMC9972845

[B80] Handler P. Cohn D. V. Dratz A. F. (1954). “Effect of parathyroid extract on renal function,” in En: Metabolic interrelations. (New York: Progress Associates), 320–332.

[B81] Heller M. Mclean F. C. Bloom W. (1950). Cellular transformations in mammalian bones induced by parathyroid extract. Am. J. Anat. 87 (3), 315–345. 10.1002/aja.1000870302 14789739

[B82] Hock J. M. Gera I. (1992). Effects of continuous and intermittent administration and inhibition of resorption on the anabolic response of bone to parathyroid hormone. J. Bone Min. Res. 7 (1), 65–72. 10.1002/jbmr.5650070110 1532281

[B83] Hruska K. A. Sugatani T. Agapova O. Fang Y. (2017). The chronic kidney disease — mineral bone disorder (CKD-MBD): Advances in pathophysiology. Bone 100, 80–86. 10.1016/j.bone.2017.01.023 28119179 PMC5502716

[B84] Hurley M. M. Okada Y. Xiao L. Tanaka Y. Ito M. Okimoto N. (2006). Impaired bone anabolic response to parathyroid hormone in Fgf2-/and Fgf2+/mice. Biochem. Biophys. Res. Commun. 341 (4), 989–994. 10.1016/j.bbrc.2006.01.044 16455048

[B85] Itoh K. Udagawa N. Matsuzaki K. Takami M. Amano H. Shinki T. (2000). Importance of membrane- or matrix-associated forms of M-CSF and RANKL/ODF in osteoclastogenesis supported by SaOS-4/3 cells expressing recombinant PTH/PTHrP receptors. J. bone mineral Res. 15 (9), 1766–1775. 10.1359/jbmr.2000.15.9.1766 10976996

[B86] Jang M-G. Lee J. Y. Yang J-Y. Park H. Kim J. H. Kim J-E. (2016). Intermittent PTH treatment can delay the transformation of mature osteoblasts into lining cells on the periosteal surfaces. J. Bone Min. Metab. 34 (5), 532–539. 10.1007/s00774-015-0707-x 26303221

[B87] Jara A. Bover J. Arnold J. (1995). Development of secondary hyperparathyroidism and bone disease in diabetic rats with renal failure. Kidney Int. 47, 1746–1751. 10.1038/ki.1995.241 7643545

[B88] Jørgensen H. S. Behets G. Viaene L. Bammens B. Claes K. Meijers B. (2022). Diagnostic accuracy of noninvasive bone turnover markers in renal osteodystrophy. Am. J. Kidney Dis. 79 (5), 667–676.e1. Epub 2021 Oct 26. PMID: 34710517. 10.1053/j.ajkd.2021.07.027 34710517

[B89] Jørgensen H. S. Ferreira A. C. D'Haese P. Haarhaus M. Vervloet M. Lafage-Proust M. H. (2022). Bone histomorphometry for the diagnosis of renal osteodystrophy: A call for harmonization of reference ranges. Kidney Int. 102 (2), 431–434. Epub 2022 May 25. PMID: 35643374. 10.1016/j.kint.2022.04.030 35643374

[B90] Jüppner H. Abou-Samra A. B. Freeman M. Kong X. F. Schipani E. Richards J. (1991). A G protein-linked receptor for parathyroid hormone and parathyroid hormone-related peptide. Science 254 (5034), 1024–1026. 10.1126/science.1658941 1658941

[B91] Kalantar-Zadeh K. Jafar T. H. Nitsch D. Neuen B. L. Perkovic V. (2021). Chronic kidney disease. Lancet 398 (10302), 786–802. Epub 2021 Jun 24. 10.1016/S0140-6736(21)00519-5 34175022

[B92] Katsimbri P. (2017). The biology of normal bone remodelling. Eur. J. Cancer Care (Engl). 26 (6), e12740. 10.1111/ecc.12740 28786518

[B93] Kendler D. L. Cosman F. Stad R. K. Ferrari S. (2022). Denosumab in the treatment of osteoporosis: 10 Years later: A narrative review. Adv. Ther. 39 (1), 58–74. Epub 2021 Nov 11. PMID: 34762286; PMCID: PMC8799550. 10.1007/s12325-021-01936-y 34762286 PMC8799550

[B94] Ketteler M. Block G. A. Evenepoel P. Fukagawa M. Herzog C. A. McCann L. (2017). Executive summary of the 2017 KDIGO chronic kidney disease–mineral and bone disorder (CKD-MBD) guideline update: what’s changed and why it matters. Kidney Int. 92 (6), 26–36. 10.1016/j.kint.2017.04.006 28646995

[B95] Ketteler M. Bover J. Mazzaferro S. ERA CKD-MBD Working Groups (2022). Treatment of secondary hyperparathyroidism in non-dialysis CKD: An appraisal 2022s. Nephrol. Dial. Transplant. 2022, gfac236–7. 10.1093/ndt/gfac236 PMC10229290 35977397

[B96] Kidney Disease: Improving Global Outcomes (KDIGO) CKD-MBD Update Work Group (2017). KDIGO 2017 clinical practice guideline update for the diagnosis, evaluation, prevention, and treatment of chronic kidney disease–mineral and bone disorder (CKD-MBD). Kidney Int. Suppl. 7, 1–59. 10.1016/j.kisu.2017.04.001 PMC6340919 30675420

[B97] Kidney Disease: Improving Global Outcomes (KDIGO)CKD–MBD Work Group (2009). KDIGO clinical practice guideline for the diagnosis, evaluation, prevention, and treatment of chronic kidney disease–mineral and bone disorder (CKD–MBD). Kidney Int. 76, S1–S130. 10.1038/ki.2009.188 19644521

[B98] Kim A. S. Girgis C. M. McDonald M. M. (2022). Osteoclast recycling and the rebound phenomenon following denosumab discontinuation. Curr. Osteoporos. Rep. 20 (6), 505–515. Epub 2022 Oct 6. PMID: 36201122; PMCID: PMC9718877. 10.1007/s11914-022-00756-5 36201122 PMC9718877

[B99] Kim J-B. Leucht P. Luppen C. A. Park Y. J. Beggs H. E. Damsky C. H. (2007). Reconciling the roles of FAK in osteoblast differentiation, osteoclast remodeling, and bone regeneration. Bone 41 (1), 39–51. 10.1016/j.bone.2007.01.024 17459803 PMC2699353

[B100] Kim M. S. Magno C. L. Day C. J. Morrison N. A. (2006). Induction of chemokines and chemokine receptors CCR2b and CCR4 in authentic human osteoclasts differentiated with RANKL and osteoclast like cells differentiated by MCP-1 and RANTES. J. Cell Biochem. 97 (3), 512–518. 10.1002/jcb.20649 16211583

[B101] Komaba H. Hamano T. Fujii N. Moriwaki K. Wada A. Masakane I. (2022). Parathyroidectomy vs cinacalcet among patients undergoing hemodialysis. J. Clin. Endocrinol. Metab. 107 (7), 2016–2025. 10.1210/clinem/dgac142 35277957

[B102] Komaba H. Taniguchi M. Wada A. Iseki K. Tsubakihara Y. Fukagawa M. (2015). Parathyroidectomy and survival among Japanese hemodialysis patients with secondary hyperparathyroidism. Kidney Int. 88, 350–359. 10.1038/ki.2015.72 25786097

[B103] Kondo H. Guo J. Bringhurst F. R. (2002). Cyclic adenosine monophosphate/protein kinase A mediates parathyroid hormone/parathyroid hormone-related protein receptor regulation of osteoclastogenesis and expression of RANKL and osteoprotegerin mRNAs by marrow stromal cells. J. Bone Min. Res. 17 (9), 1667–1679. 10.1359/jbmr.2002.17.9.1667 12211438

[B104] Levin A. Bakris G. L. Molitch M. Smulders M. Tian J. Williams L. A. (2007). Prevalence of abnormal serum vitamin D, PTH, calcium, and phosphorus in patients with chronic kidney disease: Results of the study to evaluate early kidney disease. Kidney Int. 71, 31–38. 10.1038/sj.ki.5002009 17091124

[B105] Li J. Sarosi I. Cattley R. C. Pretorius J. Asuncion F. Grisanti M. (2006). Dkk1-mediated inhibition of Wnt signaling in bone results in osteopenia. Bone 39, 754–766. 10.1016/j.bone.2006.03.017 16730481

[B106] Llach F. Bover J. (2000). “Renal osteodystrophies,” in The kidney. Editor Brenner B. M. (Philadelphia: W.B. Saunders Company).

[B107] Loots G. G. Kneissel M. Keller H. Baptist M. Chang J. Collette N. M. (2005). Genomic deletion of a long-range bone enhancer misregulates sclerostin in Van Buchem disease. Genome Res. 15 (7), 928–935. 10.1101/gr.3437105 15965026 PMC1172036

[B108] Lotinun S. Sibonga J. D. Turner R. T. (2005). Evidence that the cells responsible for marrow fibrosis in a rat model for hyperparathyroidism are preosteoblasts. Endocrinology 146 (9), 4074–4081. Epub 2005 Jun 9. PMID: 15947001. 10.1210/en.2005-0480 15947001

[B109] Lowry M. B. Lotinun S. Leontovich A. A. Zhang M. Maran A. Shogren K. L. (2008). Osteitis fibrosa is mediated by Platelet-Derived Growth Factor-A via a phosphoinositide 3-kinase-dependent signaling pathway in a rat model for chronic hyperparathyroidism. Endocrinology 149 (11), 5735–5746. 10.1210/en.2008-0134 18635661 PMC2584582

[B110] Lucas R. C. (1883). On a form of late rickets associated with albuminuria, rickets of adolescents. Lancet 121 (3119), 993–994. 10.1016/s0140-6736(02)37965-0

[B111] Lunyera J. Scialla J. J. (2018). Update on chronic kidney disease mineral and bone disorder in cardiovascular disease. Semin. Nephrol. 38 (6), 542–558. PMID: 30413250; PMCID: PMC6372293. 10.1016/j.semnephrol.2018.08.001 30413250 PMC6372293

[B112] Luo J. Yang Z. Ma Y. Yue Z. Lin H. Qu G. (2016). LGR4 is a receptor for RANKL and negatively regulates osteoclast differentiation and bone resorption. Nat. Med. 22 (5), 539–546. 10.1038/nm.4076 27064449

[B113] Luo J. Zhou W. Zhou X. Li D. Weng J. Yi Z. (2009). Regulation of bone formation and remodeling by G-protein-coupled receptor 48. Development 136 (16), 2747–2756. 10.1242/dev.033571 19605502 PMC2730404

[B114] Luxenburg C. Geblinger D. Klein E. Anderson K. Hanein D. Geiger B. (2007). The architecture of the adhesive apparatus of cultured osteoclasts: From podosome formation to sealing zone assembly. PLoS One 2 (1), e179. 10.1371/journal.pone.0000179 17264882 PMC1779809

[B115] Ma Y. L. Cain R. L. Halladay D. L. Yang X. Zeng Q. Miles R. R. (2001). Catabolic effects of continuous human PTH (1–38) *in vivo* is associated with sustained stimulation of RANKL and inhibition of osteoprotegerin and gene-associated bone formation. Endocrinology 142 (9), 4047–4054. 10.1210/endo.142.9.8356 11517184

[B116] Malluche H. H. Faugere M-C. (1986). Atlas of mineralized bone histology. Basilea, Suiza: S Karger AG.

[B117] Malluche H. H. Mawad H. W. Monier-Faugere M-C. (2011). Renal osteodystrophy in the first decade of the new millennium: Analysis of 630 bone biopsies in black and white patients. J. Bone Min. Res. 26 (6), 1368–1376. 10.1002/jbmr.309 PMC3312761 21611975

[B118] Malluche H. H. Sherman D. Meyer W. Ritz E. Norman A. W. Massry S. G. (1982). Effects of long-term infusion of physiologic doses of 1-34 PTH on bone. Am. J. Physiol. 242 (2), F197–F201. PMID: 7065136. 10.1152/ajprenal.1982.242.2.F197 7065136

[B119] Martin T. J. Sims N. A. (2005). Osteoclast-derived activity in the coupling of bone formation to resorption. Trends Mol. Med. 11 (2), 76–81. 10.1016/j.molmed.2004.12.004 15694870

[B120] Massy Z. Drueke T. (2017). Adynamic bone disease is a predominant bone pattern in early stages of chronic kidney disease. J. Nephrol. 30 (5), 629–634. 10.1007/s40620-017-0397-7 28405928

[B121] Massy Z. A. Hénaut L. Larsson T. E. Vervloet M. G. (2014). Calcium-sensing receptor activation in chronic kidney disease: Effects beyond parathyroid hormone control. Semin. Nephrol. 34 (6), 648–659. 10.1016/j.semnephrol.2014.10.001 25498383

[B122] Mazzaferro S. Pasquali M. (2021). Bone biopsy in chronic kidney disease: Still neglected and in need of revitalization. Nephrol. Dial. Transpl. 36, 202–204. 10.1093/ndt/gfaa269 33188695

[B123] McClung M. R. Bolognese M. A. Brown J. P. Reginster J. Y. Langdahl B. L. Maddox J. (2020). A single dose of zoledronate preserves bone mineral density for up to 2 years after a second course of romosozumab. Osteoporos. Int. 31 (11), 2231–2241. Epub 2020 Jul 4. PMID: 32623487; PMCID: PMC7560921. 10.1007/s00198-020-05502-0 32623487 PMC7560921

[B124] McMahon D. J. Carrelli A. Palmeri N. Zhang C. DiTullio M. Silverberg S. J. (2015). Effect of parathyroidectomy upon left ventricular mass in primary hyperparathyroidism: A meta-analysis. J. Clin. Endocrinol. Metab. 100, 4399–4407. 10.1210/jc.2015-3202 26445115 PMC4667168

[B125] Miller P. D. Adachi J. D. Albergaria B.-H. Cheung A. M. Chines A. A. Gielen E. (2022). Efficacy and safety of romosozumab among postmenopausal women with osteoporosis and mild-to-moderate chronic kidney disease. J. Bone Mineral Res. 37 (8), 1437–1445. 10.1002/jbmr.4563 PMC9544335 35466448

[B126] Moe S. Drüeke T. Cunningham J. Goodman W. Martin K. Olgaard K. (2006). Definition, evaluation, and classification of renal osteodystrophy: A position statement from kidney disease: Improving global outcomes (KDIGO). Kidney Int. 69 (11), 1945–1953. 10.1038/sj.ki.5000414 16641930

[B127] Moe S. M. Chen N. X. Newman C. L. Organ J. M. Kneissel M. Kramer I. (2015). Anti‐sclerostin antibody treatment in a rat model of progressive renal osteodystrophy. J. Bone Mineral Res. 30 (3), 499–509. 10.1002/jbmr.2372 PMC4333005 25407607

[B128] Moe S. M. Nickolas T. L. (2016). Fractures in patients with CKD: Time for action. Clin. J. Am. Soc. Nephrol. 11 (11), 1929–1931. Epub 2016 Oct 24. PMID: 27797903; PMCID: PMC5108207. 10.2215/CJN.09500916 27797903 PMC5108207

[B129] Moore C. Yee J. Malluche H. Rao D. S. Monier-Faugere M. C. Adams E. (2009). Relationship between bone histology and markers of bone and mineral metabolism in african-American hemodialysis patients. Clin. J. Am. Soc. Nephrol. 4 (9), 1484–1493. 10.2215/CJN.01770408 19713297 PMC2736690

[B130] Moysés R. M. Schiavi S. C. (2015). Sclerostin, osteocytes, and chronic kidney disease mineral bone disorder. Semin. Dial. 28 (6), 578–586. Epub 2015 Aug 19. PMID: 26288182. 10.1111/sdi.12415 26288182

[B131] Nagata Y. Imanishi Y. Tateishi T. Miyaoka D. Kurajoh M. Arnold A. (2022). Parathyroid hormone regulates circulating levels of sclerostin and FGF23 in a primary hyperparathyroidism model. J. Endocr. Soc. 6, bvac027–10. 10.1210/jendso/bvac027 35284773 PMC8907412

[B132] Nagy E. Sobh M. Abdalbary M. Elnagar S. Elrefaey R. Shabaka S. (2022). Is adynamic bone always a disease? Lessons from patients with chronic kidney disease. J. Clin. Med. 11, 7130. 10.3390/jcm11237130 36498703 PMC9736225

[B133] Naji Rad S. Deluxe L. (2022). “Osteitis fibrosa cystica,” in StatPearls (Treasure Island: StatPearls Publishing). 32644523

[B134] Nakashima T. Takayanagi H. (2011). RANKL signal and osteoimmunology. Clin. Calcium 21 (8), 1131–1140. 21814017

[B135] Naylor K. L. Garg A. X. Zou G. Langsetmo L. Leslie W. D. Fraser L. A. (2015). Comparison of fracture risk prediction among individuals with reduced and normal kidney function. Clin. J. Am. Soc. Nephrol. 10 (4), 646–653. 10.2215/CJN.06040614 25655423 PMC4386249

[B136] Neale M. (2017). Weitzmann; session: Bone as an endocrine organ; bone and the immune system. Toxicol. Pathol. 45 (7), 911–924. 10.1177/0192623317735316 29046115 PMC5749254

[B137] Nebeker H. G. Coburn J. W. (1986). Aluminum and renal osteodystrophy. Annu. Rev. Med. 37 (1), 79–95. 10.1146/annurev.me.37.020186.000455 3085581

[B138] Neer R. M. Arnaud C. D. Zanchetta J. R. Prince R. Gaich G. A. Reginster J. Y. (2001). Effect of parathyroid hormone (1-34) on fractures and bone mineral density in postmenopausal women with osteoporosis. N. Engl. J. Med. 344 (19), 1434–1441. 10.1056/NEJM200105103441904 11346808

[B139] Neto R. Pereira L. Magalhães J. Quelhas-Santos J. Martins S. Carvalho C. (2021). Sclerostin and DKK1 circulating levels associate with low bone turnover in patients with chronic kidney disease Stages 3 and 4. Clin. Kidney J. 14 (11), 2401–2408. 10.1093/ckj/sfab081 34754436 PMC8572981

[B140] Ng A. H. Omelon S. Variola F. Allo B. Willett T. L. Alman B. A. (2016). Adynamic bone decreases bone toughness during aging by affecting mineral and matrix: Adynamic bone decreases bone toughness during aging. J. Bone Min. Res. 31 (2), 369–379. 10.1002/jbmr.2702 26332924

[B141] Niall H. D. Keutmann H. Sauer R. Hogan M. Dawson B. Aurbach G. (1970). The amino acid sequence of bovine parathyroid hormone I. Hoppe Seylers Z Physiol. Chem. 351 (12), 1586–1588. 5531031

[B142] Nishida S. Yamaguchi A. Tanizawa T. Endo N. Mashiba T. Uchiyama Y. (1994). Increased bone formation by intermittent parathyroid hormone administration is due to the stimulation of proliferation and differentiation of osteoprogenitor cells in bone marrow. Bone 15 (6), 717–723. 10.1016/8756-3282(94)90322-0 7873302

[B143] Nitta K. Akiba T. Suzuki K. Uchida K. Watanabe R. Majima K. (2004). Effects of cyclic intermittent etidronate therapy on coronary artery calcification in patients receiving long-term hemodialysis. Am. J. Kidney Dis. 44 (4), 680–688. 10.1016/s0272-6386(04)00937-0 15384019

[B144] O’Brien C. A. Nakashima T. Takayanagi H. (2013). Osteocyte control of osteoclastogenesis. Bone 54 (2), 258–263. 10.1016/j.bone.2012.08.121 22939943 PMC3538915

[B145] O’Brien C. A. Plotkin L. I. Galli C. Goellner J. J. Gortazar A. R. Allen M. R. (2008). Control of bone mass and remodeling by PTH receptor signaling in osteocytes. PLoS One 3 (8), e2942. 10.1371/journal.pone.0002942 18698360 PMC2491588

[B146] Orlando M. (2020). Fibroblast growth factor 23 and the last mile. Clin. J. Am. Soc. Nephrol. 15, 1355–1357. 10.2215/CJN.13631119 32276945 PMC7480560

[B147] Pablo A. (2020). “Ureña-torres, jordi bover and martine cohen-solal. Relation between PTH and biochemical markers of MBD,” in Parathyroid glands in chronic kidney disease. Editor Covic A. (Berlin, Germany: Researchgate). 10.1007/978-3-030-43769-5_7

[B148] Parfitt A. M. Drezner M. K. Glorieux F. H. Kanis J. A. Malluche H. Meunier P. J. (1987). Bone histomorphometry: Standardization of nomenclature, symbols, and units. Report of the ASBMR histomorphometry nomenclature committee. J. Bone Min. Res. 2 (6), 595–610. 10.1002/jbmr.5650020617 3455637

[B149] Paul D. Miller M. D. Gary Hattersley PhD. Bente Juel Riis M. D. GregoryWilliams C. PhD. Edith Lau M. D. (2016). Effect of abaloparatide vs placebo on new vertebral fractures in postmenopausal women with osteoporosis: A randomized clinical trial. JAMA 316 (7), 722–733. 10.1001/jama.2016.11136 27533157

[B150] Pimentel A. Ureña-Torres P. Bover J. Luis Fernandez-Martín J. Cohen-Solal M. (2021). Bone fragility fractures in CKD patients. Calcif. Tissue Int. 108 (4), 539–550. 10.1007/s00223-020-00779-z 33219822 PMC8052229

[B151] Pimentel A. Ureña-Torres P. Zillikens M. C. Bover J. Cohen-Solal M. (2017). Fractures in patients with CKD-diagnosis, treatment, and prevention: A review by members of the European calcified tissue society and the European renal association of nephrology dialysis and transplantation. Kidney Int. 92 (6), 1343–1355. Epub 2017 Sep 28. PMID: 28964571. 10.1016/j.kint.2017.07.021 28964571

[B152] Rendina-Ruedy E. Rosen C. J. (2022). Parathyroid hormone (PTH) regulation of metabolic homeostasis: An old dog teaches us new tricks. Mol. Metab. 60, 101480. 10.1016/j.molmet.2022.101480 35338013 PMC8980887

[B153] Rhee Y. Bivi N. Farrow E. Lezcano V. Plotkin L. I. White K. E. (2011). Parathyroid hormone receptor signaling in osteocytes increases the expression of fibroblast growth factor-23 *in vitro* and *in vivo* . Bone 49 (4), 636–643. Epub 2011 Jun 25. 10.1016/j.bone.2011.06.025 21726676 PMC3167030

[B154] Richter B. Faul C. (2018). FGF23 actions on target tissues-with and without klotho. Front. Endocrinol. (Lausanne) 9, 189. 10.3389/fendo.2018.00189 29770125 PMC5940753

[B155] Rochefort G. Y. Pallu S. Benhamou C. L. (2010). Osteocyte: The unrecognized side of bone tissue. Osteoporos. Int. 21 (9), 1457–1469. 10.1007/s00198-010-1194-5 20204595

[B156] Ruffoni D. Fratzl P. Roschger P. Klaushofer K. Weinkamer R. (2007). The bone mineralization density distribution as a fingerprint of the mineralization process. Bone 40, 1308–1319. 10.1016/j.bone.2007.01.012 17337263

[B157] Sabbagh Y. Graciolli F. G. O'Brien S. Tang W. dos Reis L. M. Ryan S. (2012). Repression of osteocyte Wnt/β-catenin signaling is an early event in the progression of renal osteodystrophy. J. Bone Min. Res. 27 (8), 1757–1772. 10.1002/jbmr.1630 22492547

[B158] Salusky I. B. Coburn J. W. Brill J. Foley J. Slatopolsky E. Fine R. N. (1988). Bone disease in pediatric patients undergoing dialysis with CAPD or CCPD. Kidney Int. 33 (5), 975–982. 10.1038/ki.1988.96 3392886

[B159] Sato M. Inaba M. Yamada S. Emoto M. Ohno Y. Tsujimoto Y. (2021). Efficacy of romosozumab in patients with osteoporosis on maintenance hemodialysis in Japan; an observational study. J. Bone Min. Metab. 39 (6), 1082–1090. 10.1007/s00774-021-01253-y 34324082

[B160] Shimizu E. Nakatani T. He Z. Partridge N. C. (2014). Parathyroid hormone regulates histone deacetylase (HDAC) 4 through protein kinase A-mediated phosphorylation and dephosphorylation in osteoblastic cells. J. Biol. Chem. 289 (31), 21340–21350. 10.1074/jbc.M114.550699 24904057 PMC4118099

[B161] Shimizu E. Selvamurugan N. Westendorf J. J. Olson E. N. Partridge N. C. (2010). HDAC4 represses matrix metalloproteinase-13 transcription in osteoblastic cells, and parathyroid hormone controls this repression. J. Biol. Chem. 285 (13), 9616–9626. 10.1074/jbc.M109.094862 20097749 PMC2843211

[B162] Silva B. C. Bilezikian J. P. (2015). Parathyroid hormone: Anabolic and catabolic actions on the skeleton. Curr. Opin. Pharmacol. 22, 41–50. Epub 2015 Apr 5. PMID: 25854704; PMCID: PMC5407089. 10.1016/j.coph.2015.03.005 25854704 PMC5407089

[B163] Silva B. C. Leslie W. D. Resch H. Lamy O. Lesnyak O. Binkley N. (2014). Trabecular bone score: A noninvasive analytical method based upon the DXA image. J. Bone Min. Res. 29 (3), 518–530. 10.1002/jbmr.2176 24443324

[B164] Siris E. S. Chen Y. T. Abbott T. A. Barrett-Connor E. Miller P. D. Wehren L. E. (2004). Bone mineral density thresholds for pharmacological intervention to prevent fractures. Arch. Intern Med. 164, 1108–1112. 10.1001/archinte.164.10.1108 15159268

[B165] Siris E. S. Adler R. Bilezikian J. Bolognese M. Dawson-Hughes B. Favus M. J. (2014). The clinical diagnosis of osteoporosis: A position statement from the national bone health alliance working group. Osteoporos. Int. 25, 1439–1443. 10.1007/s00198-014-2655-z 24577348 PMC3988515

[B166] Sprague S. M. Bellorin-Font E. Jorgetti V. Carvalho A. B. Malluche H. H. Ferreira A. (2016). Diagnostic accuracy of bone turnover markers and bone histology in patients with CKD treated by dialysis. Am. J. Kidney Dis. 67, 559–566. 10.1053/j.ajkd.2015.06.023 26321176

[B167] Sprague S. M. Bellorin-Font E. Jorgetti V. Carvalho A. B. Malluche H. H. Ferreira A. (2016). Diagnostic accuracy of bone turnover markers and bone histology in patients with CKD treated by dialysis. Am. J. Kidney Dis. 67 (4), 559–566. 10.1053/j.ajkd.2015.06.023 26321176

[B168] Sturgeon C. M. Sprague S. Almond A. Cavalier E. Fraser W. D. Algeciras-Schimnich A. (2017). Perspective and priorities for improvement of parathyroid hormone (PTH) measurement A view from the IFCC Working Group for PTH. Clin. Chim. Acta 467, 42–47. 10.1016/j.cca.2016.10.016 27746210 PMC5695551

[B169] Sugatani T. Agapova O. A. Fang Y. Berman A. G. Wallace J. M. Malluche H. H. (2017). Ligand trap of the activin receptor type IIA inhibits osteoclast stimulation of bone remodeling in diabetic mice with chronic kidney disease. Kidney Int. 91, 86–95. 10.1016/j.kint.2016.07.039 27666759 PMC5530394

[B170] Sumida K. Ubara Y. Hoshino J. Mise K. Hayami N. Suwabe T. (2016). Once-weekly teriparatide in hemodialysis patients with hypoparathyroidism and low bone mass: A prospective study. Osteoporos. Int. 27 (4), 1441–1450. Epub 2015 Nov 2. PMID: 26525045. 10.1007/s00198-015-3377-6 26525045

[B171] Suzuki T. Mizobuchi M. Yoshida S. Terado N. Aoki S. Sato N. (2022). Romosozumab successfully regulated progressive osteoporosis in a patient with autosomal dominant polycystic kidney disease undergoing hemodialysis. Osteoporos. Int. 33, 2649–2652. 10.1007/s00198-022-06534-4 35980440

[B172] Takada I. Mihara M. Suzawa M. Ohtake F. Kobayashi S. Igarashi M. (2007). A histone lysine methyltransferase activated by non-canonical Wnt signalling suppresses PPAR-gamma transactivation. Nat. Cell Biol. 9 (11), 1273–1285. 10.1038/ncb1647 17952062

[B173] Tanaka K-I. Yamaguchi T. Kanazawa I. Sugimoto T. (2015). Effects of high glucose and advanced glycation end products on the expressions of sclerostin and RANKL as well as apoptosis in osteocyte-like MLO-Y4-A2 cells. Biochem. Biophys. Res. Commun. 461 (2), 193–199. 10.1016/j.bbrc.2015.02.091 25721666

[B174] Tasnim N. Dutta P. Nayeem J. Masud P. Ferdousi A. Ghosh A. S. (2021). Osteoporosis, an inevitable circumstance of chronic kidney disease: A systematic review. Cureus 13 (10), e18488. PMID: 34692259; PMCID: PMC8526087. 10.7759/cureus.18488 34692259 PMC8526087

[B175] Terauchi M. Li J. Y. Bedi B. Baek K. H. Tawfeek H. Galley S. (2009). T lymphocytes amplify the anabolic activity of parathyroid hormone through Wnt10b signaling. Cell Metab. 10 (3), 229–240. 10.1016/j.cmet.2009.07.010 19723499 PMC2751855

[B176] Torregrosa J.-V. Bover J. Portillo M. R. Parra E. G. Caravaca Francisco CasausLorenzo M-L. G. Víctor (2022). Recomendaciones de la Sociedad española de Nefrología para el manejo de las alteraciones del metabolismo óseo-mineral en los pacientes con enfermedad renal crónica: 2021 (SEN-MM). Nefrología 42 (3), 1–37. 10.1016/j.nefro.2022.03.007 18338978

[B177] Torres P. U. Bover J. Mazzaferro S. de Vernejoul M. C. Cohen-Solal M. (2014). When, how, and why a bone biopsy should Be performed in patients with chronic kidney disease. Seminars Nephrol. 34 (6), 612–625. 10.1016/j.semnephrol.2014.09.004 25498380

[B178] Tridimas A. Anna M. Marks E. (2021). Assessing bone formation in patients with chronic kidney disease using procollagen type I N-terminal propeptide (PINP): The choice of assay makes a difference. Ann. Clin. Biochem. 58 (5), 528–536. 10.1177/00045632211025567 34096326

[B179] Tsourdi E. Langdahl B. Cohen-Solal M. Aubry-Rozier B. Eriksen E. F. Guañabens N. (2017). Discontinuation of denosumab therapy for osteoporosis: A systematic review and position statement by ects. Bone 105, 11–17. Epub 2017 Aug 5. PMID: 28789921. 10.1016/j.bone.2017.08.003 28789921

[B180] Ubara Y. Tagami T. Nakanishi S. Sawa N. Hoshino J. Suwabe T. (2005). Significance of minimodeling in dialysis patients with adynamic bone disease. Kidney Int. 68 (2), 833–839. 10.1111/j.1523-1755.2005.00464.x 16014063

[B181] Ureña P. Hruby M. Ferreira A. Ang K. S. de Vernejoul M. (1996). Plasma total versus bone alkaline phosphatase as markers of bone turnover in hemodialysis patients. J. Am. Soc. Nephrol. 7 (3), 506–512. 10.1681/ASN.V73506 8704118

[B182] Ureña-Torres P. A. Vervloet M. Mazzaferro S. Oury F. Brandenburg V. Bover J. (2018). Novel insights into parathyroid hormone: Report of the parathyroid day in chronic kidney disease. Clin. Kidney J. 12 (2), 269–280. 10.1093/ckj/sfy061 30976408 PMC6452197

[B183] Vasicek T. J. McDevitt B. E. Freeman M. W. Fennick B. J. Hendy G. N. Potts J. T. Jr (1983). Nucleotide sequence of the human parathyroid hormone gene. Proc. Natl. Acad. Sci. U. S. A. 80 (8), 2127–2131. 10.1073/pnas.80.8.2127 6220408 PMC393770

[B184] Vasikaran S. Cooper C. Eastell R. Griesmacher A. Morris H. A. Trenti T. (2011). International osteoporosis foundation and international federation of clinical Chemistry and laboratory medicine position on bone marker standards in osteoporosis. Clin. Chem. Lab. Med. 49 (8), 1271–1274. 10.1515/CCLM.2011.602 21605012

[B185] Vervloet M. G. (2020). FGF23 measurement in chronic kidney disease: What is it really reflecting? Clin. Chim. Acta 505, 160–166. 10.1016/j.cca.2020.03.013 32156608

[B186] Vervloet M. G. Massy Z. A. Vincent M. B. Mazzaferro S. Cozzolino M. Ureña-Torres P. (2014). Bone: A new endocrine organ at the heart of chronic kidney disease and mineral and bone disorders. Lancet Diabetes Endocrinol. 2, 427–436. 10.1016/s2213-8587(14)70059-2 24795256

[B187] Von Recklinghausen F. D. (1891). “Die fibröse oder deformirende Ostitis, die Osteomalacie und die osteoplastische Carcinose in ihren gegenseitigen Beziehungen,” in Festschrift R virchow (Berlin: G. Reimer). 10.1055/s-0029-1235555 1756698

[B188] Wada T. Nakashima T. Hiroshi N. Penninger J. M. (2006). RANKL-RANK signaling in osteoclastogenesis and bone disease. Trends Mol. Med. 12 (1), 17–25. 10.1016/j.molmed.2005.11.007 16356770

[B189] Wan M. Yang C. Li J. Wu X. Yuan H. Ma H. (2008). Parathyroid hormone signaling through low-density lipoprotein-related protein 6. Genes Dev. 22 (21), 2968–2979. 10.1101/gad.1702708 18981475 PMC2577789

[B190] Wang Y. Nishida S. Boudignon B. M. Burghardt A. Elalieh H. Z. Hamilton M. M. (2007). IGF-I receptor is required for the anabolic actions of parathyroid hormone on bone. J. Bone Min. Res. 22 (9), 1329–1337. 10.1359/jbmr.070517 PMC10702248 17539737

[B191] Wang Z. JiangFang A. W. Chen H. (2018). Cardiac valve calcification and risk of cardiovascular or all-cause mortality in dialysis patients: A meta-analysis. BMC Cardiovasc. Disord. 18, 12. 10.1186/s12872-018-0747-y 29370754 PMC5785897

[B192] Wheater G. Elshahaly M. Tuck S. P. Datta H. K. van Laar J. M. (2013). The clinical utility of bone marker measurements in osteoporosis. J. Transl. Med. 11, 201. 10.1186/1479-5876-11-201 23984630 PMC3765909

[B193] WHO (1994). Assessment of fracture risk and its application to screening for postmenopausal osteoporosis. Report of a WHO Study Group. World Health Organ Tech. Rep. Ser. 843, 1–129. 7941614

[B194] Wijenayaka A. R. Kogawa M. Lim H. P. Bonewald L. F. Findlay D. M. Atkins G. J. (2011). Sclerostin stimulates osteocyte support of osteoclast activity by a RANKL-dependent pathway. PLoS ONE 6 (10), e25900. 10.1371/journal.pone.0025900 21991382 PMC3186800

[B195] Xiong J. Onal M. Jilka R. L. Weinstein R. S. Manolagas S. C. O’Brien C. A. (2011). Matrix-embedded cells control osteoclast formation. Nat. Med. 17 (10), 1235–1241. 10.1038/nm.2448 21909103 PMC3192296

[B196] Xiong J. Piemontese M. Thostenson J. D. Weinstein R. S. Manolagas S. C. O’Brien C. A. (2014). Osteocyte-derived RANKL is a critical mediator of the increased bone resorption caused by dietary calcium deficiency. Bone 66, 146–154. 10.1016/j.bone.2014.06.006 24933342 PMC4125539

[B197] Yajima A. Inaba M. Tominaga Y. Tanaka M. Otsubo S. Nitta K. (2013). Impact of lanthanum carbonate on cortical bone in dialysis patients with adynamic bone disease. Ther. Apher. Dialysis 17 (1), 41–48. 10.1111/1744-9987.12038 23586512

[B198] Yamamoto T. Hasegawa T. Fraitas P. H. L. Hongo H. Zhao S. Yamamoto T. (2021). Histochemical characteristics on minimodeling-based bone formation induced by anabolic drugs for osteoporotic treatment. Biomed. Res. 42 (5), 161–171. 10.2220/biomedres.42.161 34544992

[B199] Yun H. J. Ryoo S. R. Kim J. E. Choi Y. J. Park I. Shin G. T. (2020). Trabecular bone score may indicate chronic kidney disease-mineral and bone disorder (CKD-MBD) phenotypes in hemodialysis patients: A prospective observational study. BMC Nephrol. 21 (1), 299. PMID: 32711466; PMCID: PMC7382149. 10.1186/s12882-020-01944-0 32711466 PMC7382149

[B200] Zhang J. Cohen A. Shen B. Du L. Tasdogan A. Zhao Z. (2021). The effect of parathyroid hormone on osteogenesis is mediated partly by osteolectin. Proc. Natl. Acad. Sci. U. S. A. 118 (25), e2026176118. 10.1073/pnas.2026176118 34140410 PMC8237660

